# CemOrange2 fusions facilitate multifluorophore subcellular imaging in *C*. *elegans*

**DOI:** 10.1371/journal.pone.0214257

**Published:** 2019-03-26

**Authors:** Brian J. Thomas, Ira E. Wight, Wendy Y. Y. Chou, Marco Moreno, Zachary Dawson, Arielle Homayouni, Huiyan Huang, Hyori Kim, Hanna Jia, Justin R. Buland, Jennifer A. Wambach, F. Sessions Cole, Stephen C. Pak, Gary A. Silverman, Cliff J. Luke

**Affiliations:** 1 Department of Pediatrics, Washington University School of Medicine and St. Louis Children’s Hospital, St. Louis, MO, United States of America; 2 Division of Biology and Biomedical Sciences, Washington University in St. Louis, St. Louis, MO, United States of America; 3 Department of Cell Biology and Physiology, Washington University School of Medicine, St. Louis, MO, United States of America; 4 Department of Genetics, Washington University School of Medicine, St. Louis, MO, United States of America; University of North Carolina at Chapel Hill, UNITED STATES

## Abstract

Due to its ease of genetic manipulation and transparency, *Caenorhabditis elegans* (*C*. *elegans*) has become a preferred model system to study gene function by microscopy. The use of *Aequorea victoria* green fluorescent protein (GFP) fused to proteins or targeting sequences of interest, further expanded upon the utility of *C*. *elegans* by labeling subcellular structures, which enables following their disposition during development or in the presence of genetic mutations. Fluorescent proteins with excitation and emission spectra different from that of GFP accelerated the use of multifluorophore imaging in real time. We have expanded the repertoire of fluorescent proteins for use in *C*. *elegans* by developing a codon-optimized version of Orange2 (CemOrange2). Proteins or targeting motifs fused to CemOrange2 were distinguishable from the more common fluorophores used in the nematode; such as GFP, YFP, and mKate2. We generated a panel of CemOrange2 fusion constructs, and confirmed they were targeted to their correct subcellular addresses by colocalization with independent markers. To demonstrate the potential usefulness of this new panel of fluorescent protein markers, we showed that CemOrange2 fusion proteins could be used to: 1) monitor biological pathways, 2) multiplex with other fluorescent proteins to determine colocalization and 3) gain phenotypic knowledge of a human ABCA3 orthologue, ABT-4, trafficking variant in the *C*. *elegans* model organism.

## Introduction

Antibody staining techniques provide a sensitive means to visualize cellular dynamics such as protein disposition, vesicular and membrane trafficking and organellar morphogenesis and function. However, the images are static and may be distorted by fixation and permeabilization techniques. As an alternative to antibody staining, a transgene expressing a fluorescent protein (FP) fused to a protein of interest permits the assessment of cellular dynamics in real time [1]. Due to its transparency, small size and ease of genetic manipulation, *C*. *elegans* has become an ideal model system to visually study protein expression levels and subcellular dynamics within the context of a developing metazoan [2, 3]. Since Chalfie’s landmark description of using GFP reporters in *C*. *elegans* [4], a variety of spectrally variant FP tags have emerged [5]. However, their use in *C*. *elegans* has been limited due to the high levels of autofluorescence overlapping with the emission spectra of these different fluorophores [1, 6]. Moreover, the sensitivity of *C*. *elegans* to high-energy excitation wavelengths make dim FPs, such as blue fluorescent proteins, difficult to visualize in the nematode. Despite these limitations, CFP, GFP, YFP and mCherry FP reporters in *C*. *elegans* have proven utility in monitoring stress responses, cell death, redox states, cell division and serving as sensors for genetic screens [7–9].

As human pathological variants of unknown significance (VUS) are discovered, insight into their activity is gained by assessing their functions in model systems, like *C*. *elegans* [2, 3, 10, 11]. For example, by generating wild-type and VUS FP fusions, and expressing them in *C*. *elegans* strains harboring different FP tagged subcellular structures or organelles, multiplexing imaging studies can show a change in the disposition of the VUS (e.g., aggregation, change in subcellular localization) and confirm pathogenicity. *C*. *elegans* FP-tagged organelle makers, such as those for lysosomes and lysosome-related organelles (LRO’s), endoplasmic reticulum (ER) and autophagosomes already exist [12–16]. However, most of these markers contain the commonly used fluorophores (e.g. GFP and mCherry), making multiplexing with another FP that has overlapping excitation/emission (*Ex/Em*) spectra (e.g. YFP or mKate2) impractical. To circumvent this, newer FPs with a wider variety of *Ex/Em* spectra are now available (Table 1) [1, 17] which are more versatile, especially when coupled with non-conventional imaging technologies for enhanced spectral separation, such as supercontinuum white light lasers [18].

**Table 1:** Reported fluorescence characteristics of the fluorescent proteins used in this study.

fluorescent protein	*Ex*_max_	*Em*_max_	EC (M^-1^ cm^-1^)	QY	brightness	pKa	lifetime (ns)	photo-stability (s)	maturation (mins)
EGFP	488	507	55,900	0.6	33.5	6.0	2.6	174	25
EYFP	513	527	83,400	0.6	50.9	6.9	3.1	60	unknown
mOrange2	549	565	58,000	0.6	34.9	6.5	2.7	228	270
mCherry	587	610	72,000	0.2	15.8	4.5	1.4	68	15
mKate2	588	633	62,500	0.4	25.0	5.4	2.5	84	20
mNeptune2.5	599	643	95,000	0.2	22.8	5.8	unknown	unknown	26
mCardinal	604	659	87,000	0.2	16.5	5.3	unknown	730	27

The excitation (*Ex*) and emission (*Em*) maxima, extinction coefficients (EC), quantum yields (QY), brightness, pKa, fluorescence lifetime, photostability and maturation time of the indicated fluorescent proteins are reported on the fluorescent protein database (https://www.fpbase.org).

The goal of this work was to develop a toolbox of subcellular organelle markers with a fluorophore that could be spectrally separable from GFP, YFP and far-red fluorescent proteins, such as mKate2. Using expression vectors with multiple subcellular organelle targeting proteins or motifs fused with CemOrange2, we show that this FP was directed to the correct subcellular location in intestinal cells. Moreover, we show that transgenic animals expressing CemOrange2 can be multiplexed with other fluorophores to study different biological pathways.

## Materials and methods

### Expression plasmids

All amplifications were performed using the Q5 high fidelity or Phusion high fidelity PCR kits (NEB, Ipswich, MA). Restriction enzymes used for cloning procedures were purchased from New England Biolabs (NEB).

To generate P_*nhx-2*_ *CemOrange2*, P_*nhx-2*_ *CemNeptune2*.*5*, and P_*nhx-2*_ *CemCardinal2* constructs (pJR2956, pJR2955 and pJR2953), minigene blocks with three synthetic introns (gblock) containing *C*. *elegans* codon optimized version of mOrange2 (CemOrange2), mNeptune2.5 (CemNeptune2.5) and mCardinal2 (CemCardinal2) with additional N- and C-terminal restriction sites were synthesized (IDT, Skokie, IL) and sub-cloned into the NheI/SacI restriction sites of the canonical expression vector, pPD49.26 [17, 19]. A 2kb *nhx-2* promoter fragment was amplified (S1 Table, primer set 1) and ligated into the HindIII/XbaI restriction sites to yield the final constructs: pJR2956, pJR2955, and pJR2953, respectively.

To generate expression constructs of cloned fragments the NEBuilder HiFi DNA Assembly Cloning Kit was used (NEB). For N-terminus CemOrange2 fusions, fragments were cloned into the KasI restriction site and for C-terminus protein fusions fragments were cloned into the NheI restriction site of pJR2956. P_*nhx-2*_*sqst-1*::*CemOrange2* (pBT3037), P_*nhx-2*_*aqp-1*::*CemOrange2* (pBT3102), P_*nhx-2*_*lmn-1*::*CemOrange2* (pBT3035), P_*nhx-2*_*lmp-1*::*CemOrange2* (pBT2999), P_*nhx-2*_*aman-2*::*CemOrange2* (pLS2965), and P_*nhx-2*_*glo-1*::*CemOrange2* (pBT3038) were generated by amplifying the *sqst-1* (S1 Table, primer set 2), *aqp-1* (S1 Table, primer set 3), *lmn-1* (S1 Table, primer set 9), *lmp-1* (S1 Table, primer set 11), *aman-2* (S1 Table, primer set 15), and *glo-1* (S1 Table, primer set 16) genomic DNA fragment and ligating the fragment into the NheI site of P_*nhx-2*_ *CemOrange2* (pJR2956), respectively. P_*nhx-2*_*CemOrange2*::*lgg-1* (pBT3043), P_*nhx-2*_*CemOrange2*::*cup-5* (pBT3011), P_*nhx-2*_*CemOrange2*::*rab-5* (pBT3007), P_*nhx-2*_*CemOrange2*::*rab-7* (pBT3014), P_*nhx-2*_*CemOrange2*::*tram-1* (pBT3000), and P_*nhx-2*_*CemOrange2*::*pisy-1* (pBT3003) were generated by amplifying the *lgg-1* (S1 Table, primer set 4), *cup-5* (S1 Table, primer set 8), *rab-5* (S1 Table, primer set 12), *rab-7* (S1 Table, primer set 13), *tram-1* (S1 Table, primer set 14), and *pisy-1* (S1 Table, primer set 17) genomic DNA fragment and ligating the fragment into the KasI site of P_*nhx-2*_ *CemOrange2*, respectively.

Mitochondrial targetting (^mt^), peroxisome targeting peptide (SKL) and nuclear localization sequences (NLS) were inserted into the P_*nhx-2*_*CemOrange2* expression construct using the Q5 site-directed mutagenesis kit (NEB). P_*nhx-2*_^*mt*^*CemOrange2* (pBT3024) was generated using primer set 5 (S1 Table). P_*nhx-2*_*NLS*^*SV-40*^::*CemOrange2*::*NLS*^*egl-13*^ (pBT3047) was generated using primer sets 6 (SV-40) and 7 (*egl-13*) (S1 Table). P_*nhx-2*_*CemOrange2*::*SKL* (pBT3019) was generated using primer set 10 (S1 Table).

To generate P_*nhx-2*_*sGFP*::*KDEL* (pOL2184), the *nhx-2* promoter was amplified (S1 Table, primer set 1) and sub-cloned into the HindIII/XbaI sites of pPD95.85 to create P_*nhx-2*_*sGFP* (pAV1771). The stop codon of GFP was mutated by Quikchange site-directed mutagenesis (S1 Table, primer set 18) to the ER retention motif, KDEL, yielding the final construct, pOL2184.

To generate P_*nhx-2*_*mKate2 (*pBC2370), mKate2 was amplified from pmKate2 (Evrogen, RU) using primer set 21 (S1 Table). A 2kb *nhx-2* promoter fragment was amplified (S1 Table, primer set 1) and ligated into the HindIII/XbaI restriction sites of the canonical expression vector, pPD49.26 to yield the final constructs pBC2370. To generate P_*nhx-2*_*mKate2*::*lgg-1* (pME2707-pME2710), additional restrictions sites were added to P_*nhx-2*_*mKate2* (S1 Table, primer set 22) and *lgg-1* genomic DNA was amplified (S1 Table, primer set 23) and ligated into the PstI/SacI restriction sites to yield the final constructs pME2707-pME2710.

To generate P_*nhx-2*_*abt-4*::*mKate2* (pBT3111), *abt-4* genomic DNA was amplified using primer set 19 (S1 Table) and ligated into the NheI site of P_*nhx-2*_*mKate2*. To generate P_*nhx-2*_*abt-4*^*L162P*^::*mKate2* (pBT3114), pBT3111 was mutated with primer set 20 (S1 Table) using the Q5 site-directed mutagenesis kit.

### *C*. *elegans* strains and culture conditions

A list of worm strains and genotypes, along with the figures they correspond to is given in S2 Table for reference. All injection mixes were made to a final total DNA concentration of 100–150 ng/μl using pBluescript SK- (Agilent Technologies, Santa Clara, CA) as carrier DNA. The strain VK2266 (*vkEx2266[*P_*nhx-2*_*mKate2*::*lgg-1;*P_*myo-2*_*GFP]*) was generated by co-injecting 80 ng/μl P_*nhx-2*_*mKate2*::*lgg-1* with 5 ng/μl of P_*myo-2*_*GFP*. The strains VK2664, VK2666, VK2671, VK2674, VK2688, VK2700, VK2702, VK2728, VK2733, VK2738, VK2755, VK2756, VK2757 and VK2883 were generated by co-injecting 50 ng/μl of the expression plasmids P_*nhx-2*_*CemOrange2*::*tram-1*, P_*nhx-2*_*CemOrange2*::*rab-7*, P_*nhx-2*_*CemOrange2*::*rab-5*, P_*nhx-2*_*CemOrange2*::*pisy-1*, P_*nhx-2*_*CemOrange2*::*cup-5*, P_*nhx-2*_*CemOrange2*::*SKL*, P_*nhx-2*_^*mt*^*CemOrange2*, P_*nhx-2*_*NLS*^*SV-40*^::*CemOrange2*::*NLS*^*egl-13*^, P_*nhx-2*_*sqst-1*::*CemOrange2*, P_*nhx-2*_*CemOrange2*::*lgg-1*, P_*nhx-2*_*CemOrange2*, P_*nhx-2*_*CemCardinal2*, P_*nhx-2*_*CemNeptune2*.5, and P_*nhx-2*_*aqp-1*::*CemOrange2*, respectively, with 5 ng/μl P_*myo-2*_*GFP* (pPD118.33, a kind gift from Dr. Andrew Fire). The strain VK2620 was generated by co-injecting 20 ng/μl of the expression plasmid P_*nhx-2*_*aman-2*::*CemOrange2* with 5 ng/μl P_*myo-2*_*GFP*. The strains VK2697, VK2734, VK2735 were generated by co-injecting 10 ng/μl of the expression plasmids P_*nhx-2*_*lmp-1*::*CemOrange2*, P_*nhx-2*_*lmn-1*::*CemOrange2*, and P_*nhx-2*_*glo-1*::*CemOrange2*, respectively, with 5 ng/μl P_*myo-2*_*GFP*. The strains VK2748 and VK3007 were generated by co-injecting 15 ng/μl of P_*nhx-2*_*GFP*::*KDEL* with 35 ng ng/μl of P_*nhx-2*_*CemOrange2*::*tram-1* and P_*nhx-2*_*CemOrange2*::*pisy-1*, respectively. The strain VK2838 was generated by co-injecting 50 ng/μl P_*nhx-2*_*CemOrange2*::*SKL* with 5 ng/μl P_*myo-2*_*GFP* into VS11 [20]. The strains VK3160 and VK3161 were generated by co-injecting with 15 ng/μl P_*myo-2*_*CemOrange2*::*tram-1* with 15 ng/μl P_*nhx-2*_*abt-4*::*mKate2* and P_*nhx-2*_*abt-4*^*L162P*^::*mKate2*, respectively. The strains VK2697 and VK2734 were integrated spontaneously. The strains VK2288 (*vKIs2288[*P_*nhx-2*_*mKate2*::*lgg-1;*P_*myo-2*_*GFP]*), VK2797 (*vkIs2797[*P_*nhx-2*_*CemOrange2*::*rab-5;*P_*myo-2*_*GFP]*), VK2799 (*vkIs2799[*P_*nhx-2*_*glo-1*::*CemOrange2;*P_*myo-2*_*GFP]*), VK2807 (*vkIs2807[*P_*nhx-2*_*aman-2*::*CemOrange2;*P_*myo-2*_*GFP]*), VK2815 (*vkIs2815[*P_*nhx-2*_*CemOrange2*::*rab-7;*P_*myo-2*_*GFP]*), VK2877, and VK2878 were integrated via X-ray irradiation (*vide infra*) and derived from the strains VK2266, VK2671, VK2735, VK2620, VK2666, VK2728, and VK2738, respectively. The strain VK2617 (*vkIs2617[*P_*nhx-2*_*sGFP*::*ATZ;*P_*nhx-2*_*mKate2*::*lgg-1*;P_*myo-2*_*GFP*;P_*myo-2*_*mCherry]*) was generated by crossing males from strain VK1882 [21] with hermaphrodites from strain VK2288. The strain VK2749 was generated by crossing males from strain VK2697 with hermaphrodites from strains VK2617. The worm strains VK2881 and VK2882 were generated by crossing males from strain VK2799, with hermaphrodites from strains VS17 and RT258, respectively [20, 22]. All strains were obtained from the *Caenorhabditis* Genetic Center (CGC) unless otherwise stated. Males were generated by heat shocking at 27°C for 18 hrs or 30°C for 6 hrs. Animals were routinely cultured at 20°C on nematode growth media (NGM) plates seeded with *E*. *coli* strain OP50 unless otherwise stated.

### Transgene integration

Ten transgenic adult animals were placed on 2 separate, 100 mm NGM source plates and allowed to lay eggs for 3 days. One-hundred L4s from the source plates were transferred to one, 100 mm NGM plate. Animals were exposed to 35 Gy radiation using an X-ray irradiator. Animals were allowed to recover for 2 hrs at room temperature or 16°C overnight. Three irradiated L4 animals were placed on 30 separate, 100 mm NGM plates and grown to starvation (~7–10 days). Animals were collected in 1 ml of PBS and 10 μl was placed on 30 separate, 100 mm NGM plates. These plates were grown to starvation (~5–7 days), washed with 1 ml of PBS and 10 μl of the wash was placed onto 30 separate, 60 mm NGM plates seeded with OP50 and allowed to grow for 2 days. Six transgenic animals from each plate were transferred to 6 separate, 35 mm NGM plates and grown for 3 days. Animals that had 100% transmission of the transgenic marker were considered integrated and stocked.

### *E*. *coli* OP50 preparation

A single colony of *E*. *coli* (OP50) was placed in 5 ml LB broth and incubated at 37°C with shaking (250 rpm) overnight. This culture was then added to 500 ml of LB broth and incubated at 37°C with shaking until reaching an OD_600_ = 0.75–1.0. The bacteria were collected by centrifugation and washed once with 1:10 original volume of PBS and resuspended in 1:25 original volume in PBS. An equal volume of 50% glycerol was added for long-term storage at -80°C. After thawing, the bacteria were concentrated by centrifugation, washed and re-suspended in 1:25 original volume in PBS.

### Autophagic flux assay

Twenty late L4 animals were placed into 100 μl PBS supplemented with OP50 and the following conditions: no treatment (diluent control), 25 μM fluphenazine (Millipore-Sigma, St. Louis, MO), or 10 mM 3-methyladenine (3-MA; Millipore-Sigma). The animals were incubated for 16 hrs at RT and recovered on an NGM plate for 30 min. Animals were transferred to a 35 mm coverglass bottom petri dish (MatTek, Ashland, MA) for confocal imaging.

### Lysosomal membrane permeability (LMP) assay

Twenty adult animals were placed in a 10 μl solution of either PBS alone, 5% *tert*-butyl hydroperoxide (*t*-BOOH; Millipore-Sigma) or 500 mM Leu-Leu-OMe (LLOMe; Bachem Torrance, CA) in PBS. Animals were incubated for 15 min and transferred to a 35 mm coverglass bottom petri dish (MatTek) for confocal imaging.

### Intracellular organelle labeling

LysoTracker Deep Red (LTDR; Life Technologies Corp, Carlsbad, CA) were diluted to 1 μM in PBS. About 20–30 adult stage animals were placed in the solution and incubated at room temperature for 30 min. To chase excess dye prior to imaging, animals were placed on a fresh NGM plate seeded with OP50 for 15 min.

MitoTracker Deep Red (Life Technologies Corp) was resuspended in DMSO to a stock concentration of 1.25 mM. Prior to use, the stock solution was diluted to 500 nM in PBS. About 20–30 adult stage animals were placed in 10 μl staining solution and incubated at room temperature for 30 min. To chase excess dye prior to imaging, animals were placed on a fresh NGM plate seeded with OP50 for 15 min.

Bovine serum albumin (BSA)-Alexa Fluor conjugate (Life Technologies Corp) was resuspended in PBS to a concentration of 5 mg/ml. About 20–30 adult stage animals were placed in 10 μl BSA and 5 μl OP50 and incubated for 16hrs. To chase excess dye prior to imaging, animals were placed on a fresh NGM plate seeded with OP50 for 60 min.

BODIPY FL C_5_-Ceramide (C5 FL) complexed to BSA (Life Technologies Corp) was resuspended in water to a stock concentration of 500 μM. Prior to use, the stock solution was diluted to 50 μM with PBS supplemented with OP50. About 20–30 adult stage animals were placed in 10 μl staining solution and incubated at room temperature for 1hr. To chase excess dye prior to imaging, animals were placed on a fresh NGM plate seeded with OP50 for 30 min.

CellMask Deep Red (Life Technologies Corp) plasma membrane stain was diluted 1:100 in PBS. About 20–30 adult stage animals were placed in 10 μl staining solution and incubated at room temperature for 1 hr. To chase excess dye prior to imaging, animals were placed on a fresh NGM plate seeded with OP50 for 30–60 min.

### Microscopic imaging and analysis

To prepare animals for imaging, 6–10 μl of 100 mM NaN_3_ (Millipore-Sigma) in PBS was placed on the middle of a 35 mm coverglass bottom dish (MatTek). Approximately, 10–15 adult stage animals were transferred to the NaN_3_ solution and covered with a 12 mm circular coverslip and then a 25 mm square coverslip.

Confocal images were taken with a Lecia SP8X tandem scanning confocal microscope with a white light laser using either a 40x 1.3 NA or 63x 1.4 NA oil PlanApo objective over ≥20 *z-*planes and a pinhole size of 1.00 (Leica Microsystems, Buffalo Grove, IL). Images were displayed as single XY planes, except nuclear markers which were displayed as maximum intensity projections. Images were rendered and analyzed using LASX (Leica Microsystems) and Volocity (v6.3; Quorum Technologies, CAN) software.

Spectral data were obtained using a supercontinuum white light laser and prism-based spectral detector. Excitation spectra were determined by measuring the intensity of the sample while exciting every 3–5 nm. Sample excitation was started ~100 nm lower of reported excitation maximum (*Ex*_*max*_), up to a detection range (~40–50 nm higher than the emission maxima (*Em*_*max*_)). The *Em* spectra were determined by exciting the sample 50–100 nm lower than the *Ex*_*max*_ and measuring in a range of 20 nm in 5 nm steps.

Colocalization and quantification of data were obtained using the Volocity cellular imaging and analysis software (v6.3; Quorum Technologies). Either Pearson correlation or Manders correlation coefficient (MCC) was used to assess the positional relationship between two objects [23]. Colocalization and quantification of vesicular or punctate structures were obtained using the Volocity Colocalization or Volocity Quantification modules, respectively, with thresholding of fluorescence intensity and size to remove background noise. A minimum of 5 individual animals was used in the analysis.

### Statistical analyses

Two-tailed (heteroscedastic) t-tests were performed on quantified data using Microsoft Excel. Experiments were repeated at least two times to ensure reproducibility.

## Results

### CemOrange2 excitation and emission spectra *in vivo*

**Fig 1:**
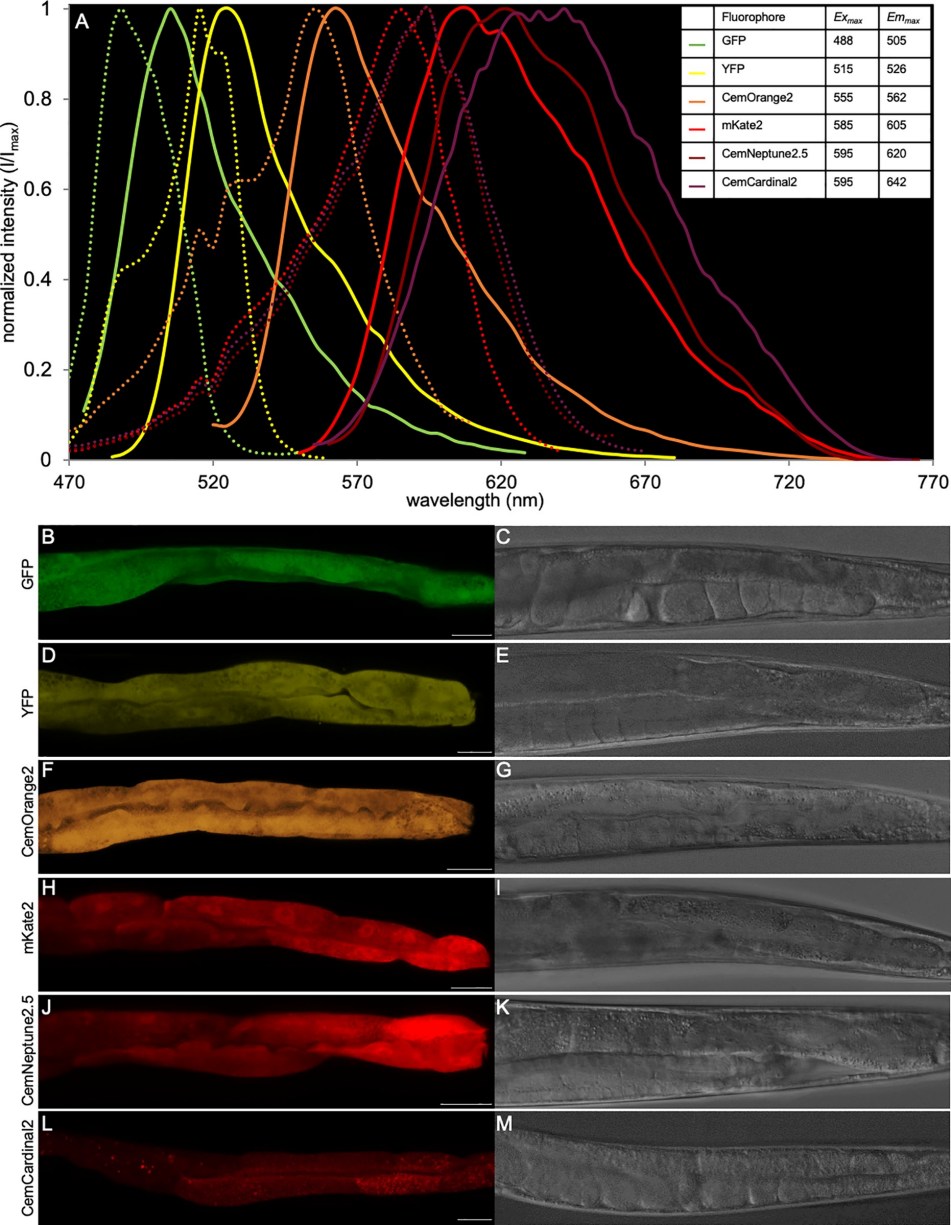
Excitation and emission spectra and cytosolic expression patterns of fluorescent proteins in *C*. *elegans*.

Detectable differences in the *Ex*/*Em* spectra of individual FPs permit the visualization of multiple fluorophores within the same physical space (Table 1). Three FPs with different spectra, GFP (*Ex*488 nm/*Em*505 nm), YFP(*Ex*515 nm/*Em*526 nm) and mKate2(*Em*585 nm/*Ex*605 nm) [24–26], have been utilized in *C*. *elegans* within our laboratory [27, 28]. To broaden our repertoire for multiplexing, we synthesized minigene gblocks for mOrange2, mNeptune2.5 and mCardinal2 [17, 29]. To enhance expression of these FPs in *C*. *elegans*, we codon optimized the cDNA sequence and introduced 3 synthetic introns using the *C*. *elegans* Codon Adapter software, with a Codon Adaption Index of 1.0 (https://worm.mpi-cbg.de/codons/cgi-bin/optimize.py) [19, 30]. The *C*. *elegans* (Ce) optimized CemOrange2, CemNeptune2.5 and CemCardinal2 gblocks were ligated into a backbone expression vector containing the intestinal promoter *nhx-2* in pPD49.26 [31, 32]. Correct plasmid construction was confirmed by DNA sequencing. These expression plasmids, along with a co-injection marker (P_*myo-2*_GFP), were introduced to the germline of N2 (wild-type) animals via microinjection. Individual transgenic animals were selected using the co-injection marker, discernable via a fluorescent stereoscope and placed onto single plates. Lines that passed the marker onto the second generation were selected for imaging and spectral analysis. The *Ex*_*max*_*/Em*_*max*_ spectra in the intestinal cytosol were determined using a confocal laser-scanning microscope equipped with a supercontinuum white light laser and prism-based spectral detector. The *Ex*_*max*_*/Em*_*max*_ were: 1) GFP, *Ex*488 nm/*Em*505 nm; 2) YFP, *Ex*515 nm/*Em*526 nm; 3) CemOrange2, *Ex*555 nm/*Em*562 nm; 4) mKate2, *Ex*585 nm/*Em*605 nm; 5) CemNeptune2.5, *Ex*595 nm/*Em*620 nm; and 6) CemCardinal2, *Ex*595 nm/*Em*642 nm (Fig 1A). Spectral characteristics show that the *Ex*_*max*_*/Em*_*max*_ spectra of CemOrange2 were between those of GFP, YFP and mKate2. CemNeptune2 and CemCardinal2 had *Ex*_*max*_ spectra shifted ~10 nm higher than that of mKate2 and *Em*_*max*_ spectra shifted ~15 nm and ~37 nm higher than that of mKate2, respectively (Fig 1A). Moreover, CemOrange2 was distinct from autofluoresence found in wild-type N2 animals (S2 Fig) These data suggest that CemOrange2 could be utilized for multifluorophore imaging with other commonly used FPs.

Excitation and emission spectra of fluorescent proteins (FPs) expressed within the cytoplasm of *C*. *elegans* intestinal cells: GFP (green), YFP (yellow), CemOrange2 (orange), mKate2 (red), CemNeptune2 (dark red), CemCardinal2 (magenta) FPs were expressed from transgenes driven by the intestinal specific promoter, P_*nhx-2*_ (A). Dotted and solid lines represent excitation and emission curves, respectively. Spectral scans in both the excitation (470 nm-670 nm) and emission (480 nm-770 nm) wavelengths were obtained using a supercontinuum white light laser and spectral detectors on a confocal microscope. Intensity was normalized to the maximum fluorescence intensity value (I/I_max_). The inset table indicates the maximal excitation (*Ex*_*max*_) and emission wavelengths (*Em*_*max*_) determined for each fluorophore. (B-M) Representative confocal maximum intensity projections of transgenic animals expressing a single FP using the *Ex*_*max*_ and *Em*_*max*_ (± 20 nm) determined from Fig 1A. Fluorescent and DIC (a single XY-plane) images are displayed in the left and right columns, respectively. Scale bar = 25 μm.

Different FPs exhibit a range of characteristics (Table 1), such as brightness, aggregation and cellular toxicity, depending on the cell or organism in which they are expressed [33]. Interestingly, the three related far-red FPs, mKate2, CemNeptune2.5 and CemCardinal2, had lower *Em*_max_ *in vivo* (Fig 1A) than previously reported. Transgenic animals expressing different cytosolic FPs within the intestine were visualized qualitatively using confocal laser-scanning microscopy at the previously determined *Ex*_*max*_*/Em*_*max*_. GFP, YFP, CemOrange2 and mKate2 were bright, diffuse and uniform (Fig 1B, 1D, 1F and 1H). CemNeptune2.5 expression was diffuse but non-uniform within the cytosol (Fig 1J). CemCardinal2 was neither bright nor uniform in its distribution and exhibited punctate structures suggestive of FP aggregation or concentration within a subcellular structure (Fig 1L). Corresponding DIC images confirmed expression was restricted to intestinal cells. (Fig 1C, 1E, 1G, 1I, 1K and 1M). Taken together, these data suggest that CemOrange2 would be the FP of choice for subcellular imaging.

### CemOrange2 fused to targeting motifs or proteins localized to subcellular structures and organelles

CemOrange2 exhibited *Ex*_*max*_*/Em*_*max*_ spectra that were distinguishable from those of GFP, YFP and mKate2. Also, CemOrange2 expressed in intestinal cells yielded a diffuse cytoplasmic pattern (i.e., no evidence of aggregation). Taken together, these characteristics suggested that this FP would facilitate multiplexing with unknown FP-tagged proteins by targeting known subcellular structures. Thus, the P_*nhx-2*_*CemOrange2* construct (pJR2945) was used to generate a transgene expressing the FP fused to different targeting motifs or a *C*. *elegans* protein known to localize to specific organelles/structures (Table 2). These structures included the nuclear lamina [34]; the rough ER [15]; the Golgi [35]; early and late endosomes [36]; the lysosome [12, 14]; autophagosomes [16]; ubiquitinylated cargos [37, 38]; lysosomal related organelles (LRO) [13, 39]; the apical, lateral and basal plasma membrane [40]; the nucleus [41]; the mitochondrial matrix [42]; and peroxisomes [43]. All genes were amplified by PCR from wild-type genomic DNA using gene-specific primers and cloned in frame with CemOrange2 at either the N- or C-termini (Table 2). Subcellular targeting subsequences were introduced at either the N- or C-termini by site-directed mutagenesis. The identities of all constructs were confirmed by DNA sequencing.

**Table 2:** *C*. *elegans* proteins and target peptides.

protein/ target peptide	human homolog (best BLASTP match)^$^	organelle	reference
TRAM-1	TRAM1 (translocating chain-associated membrane protein 1)	rough endoplasmic reticulum	[15]
PISY-1	CDIPT (isoform 1 of CDP-diacylglycerol—inositol 3-phosphatidyltransferase)	endoplasmic reticulum (some golgi structures)	[15]
RAB-7	RAB7A (ras-related protein Rab-7a)	endosome (late)	[36]
RAB-5	RAB5B (ras-related protein Rab-5B)	endosome (early)	[36]
LMP-1	LAMP1 (lysosomal associated membrane protein 1)	lysosome	[14]
CUP-5	MCOLN3 (isoform 1 of Mucolipin-3)	lysosome	[12]
AMAN-2	MAN2A1 (alpha-mannosidase 2)	Golgi	[35]
GLO-1	RAB32 (ras-related protein Rab-32)	lysosome-related organelle	[13]
SKL*		peroxisome	[43]
mt^#^		mitochondria	[42]
LGG-1	GABARAP (gamma-aminobutyric acid receptor-associated protein)	cytoplasmic/ autophagosome	[16]
SQST-1	SQSTM1 (sequestosome-1)	cytoplasmic/ autophagosome	[44]
LMN-1	LMNB1 (lamin B1)	nuclear lamina	[34]
NLS^**†**^		nucleus	[41]
AQP-1	AQP10 (aquaporin 10)	plasma membrane	[40]

^$^ www.wormbase.org

*Peroxisome target sequence = SKL

^#^mt (mitochondria target sequence) = MLSLRQSIRFFKPATRTLCSSRTLL

^**†**^NLS^SV-40^ = MAPKKKRKV; NLS^egl-13^ = MSRRRKANPTKLSENAKKLAKEVEN

**Fig 2:**
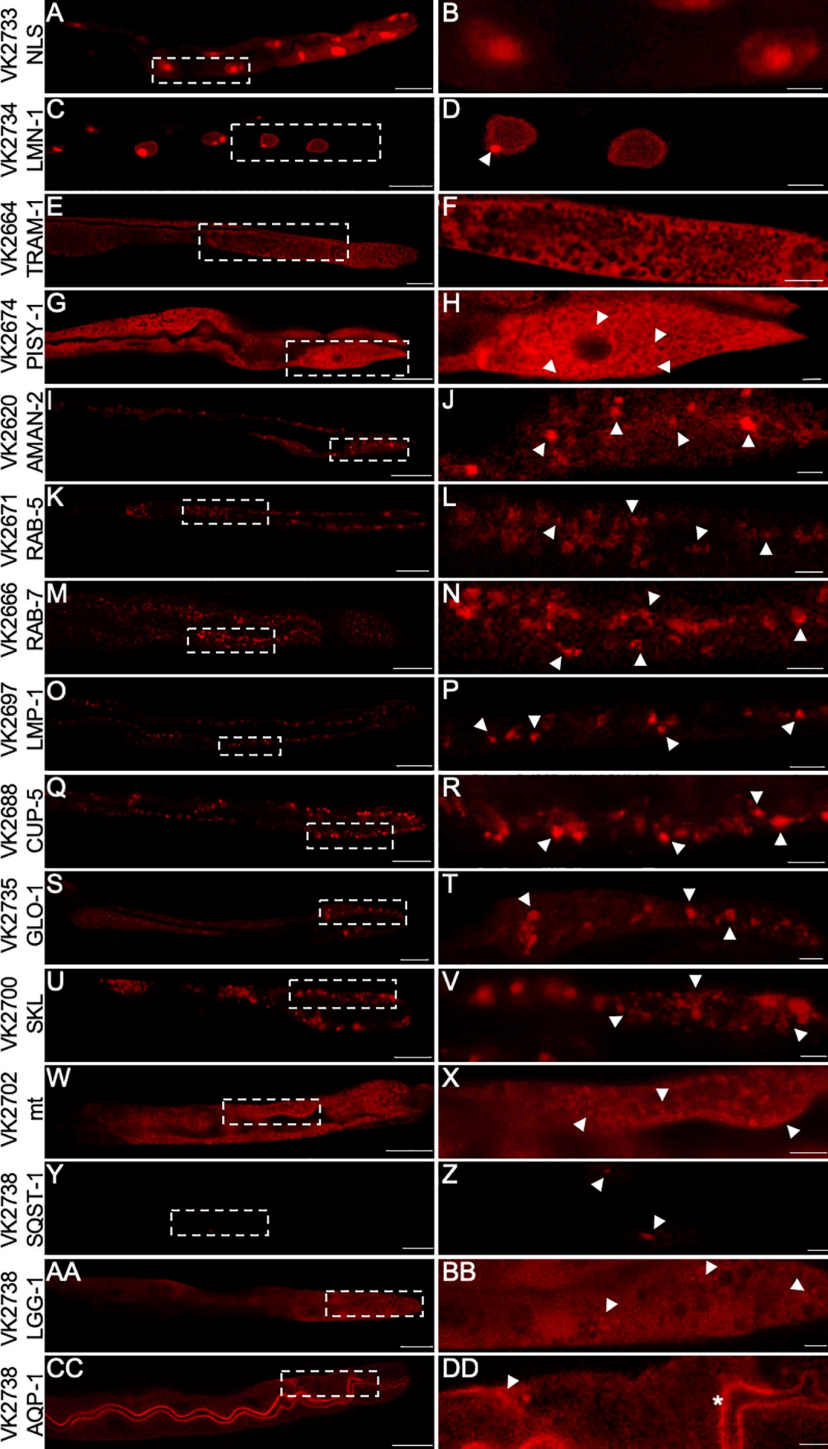
CemOrange2 subcellular expression patterns within *C*. *elegans*. Representative confocal images of transgenic *C*. *elegans* strains expressing CemOrange2 fused with: the N-terminal SV-40 and C-terminal *egl-13* nuclear localization signals (A, B); LMN-1, a nuclear lamin (C, D); TRAM-1, a rough ER protein (E, F); PISY-1, ER and Golgi protein (G, H); AMAN-2, a Golgi protein (I, J); RAB-5, an early endosomal protein (K, L); RAB-7, a late endosomal protein (M, N); LMP-1 and CUP-5, lysosomal membrane proteins (O-R); GLO-1, lysosomal related organelle protein (LRO) (S, T); SKL, a C-terminal peroxisomal targeting peptide (U, V); *mt*, an N-terminal mitochondrial target peptide (W, X); SQST-1 and LGG-1, autophagosome-related proteins (Y-BB); and AQP-1, an apical and basolateral plasma membrane protein (CC, DD). All transgenes were expressed using the intestinal specific promoter, P_*nhx-2*_. Images were collected over >20 *z*-planes using a 40x PlanApo oil immersion objective (N.A. 1.3) at wavelengths *Ex*555 nm/*Em*565-590 nm. Either a maximum intensity projection (A—D) or a single XY-plane (E-DD) of the CemOrange2 expression are shown. The dashed box demarks a single intestinal cell to highlight the subcellular expression pattern shown in the right column (B, D, F, H, J, L, N, P, R, T, V, X, Z, BB, DD). Scale bars = 25 μm (left column) or 5 μm (right column). See text for description of arrowheads and asterisk.

The subcellular localizations of the CemOrange2 fusion proteins in transgenic wild-type animals were visualized using confocal laser-scanning microscopy with an *Ex*555 nm/*Em*565-590 nm. The nucleus marker, NLS^SV-40^::CemOrange2:: NLS^egl-13^, was restricted to the nucleus (Fig 2A and 2B). The nuclear envelope marker, LMN-1::CemOrange2, showed characteristic highlighting of the nuclear lamina (Fig 2C and 2D). However, there was evidence of areas of CemOrange2 accumulation on the lamina, possibly due to overexpression artifact (white arrowhead). The ER markers, CemOrange2::TRAM-1 and CemOrange2::PISY-1, respectively, showed bright reticular fluorescence characteristic of the ER (Fig 2E and 2F and Fig 2G and 2H, respectively). The golgi localizing protein, AMAN-2::CemOrange2, showed punctate structures (~10-20/cell) characteristic of Golgi stacks (Fig 2I and 2J, white arrowheads). Similar to previous studies, CemOrange2::RAB-5 (Fig 2K and 2L) and CemOrange2::RAB-7 (Fig 2M and 2N) showed multiple sized clusters of puncta, consistent with endosomes (white arrowheads; RAB-5^+^ early and RAB-7^+^ late endosomes, respectively, cannot be distinguished under these conditions) [13, 36, 45]. Unlike previous studies, the lysosome marker, LMP-1::CemOrange2 showed discrete and variably sized punctate structures (Fig 2O and 2P, white arrowheads) [46, 47]. Similar to previously published reports, the lysosome marker, CUP-5::CemOrange2 also showed discrete punctate structures in the cytoplasm (Fig 2Q and 2R, white arrowheads) [12]. The lysosome-related organelle (LRO) marker, GLO-1::CemOrange2, demonstrated punctate structures that appeared qualitatively larger than those structures demarcated by the lysosome makers (Fig 2S and 2T, white arrowheads). The peroxisome marker, CemOrange2::SKL, showed multiple clusters of small puncta (Fig 2U and 2V, white arrowheads). The clusters of puncta localized close to basolateral membrane. The mitochondrial probe, ^mt^CemOrange2, showed densely packed small, oblong structures, that are characteristic of mitochondria (Fig 2W and 2X, white arrowheads).

The selective autophagy receptor, SQST-1::CemOrange2, is a diffuse cytosolic protein with few puncta detectable at lower resolution under normal conditions (Fig 2Y and 2Z, white arrowheads). Similarly, the autophagy marker, CemOrange2::LGG-1, also showed mainly a cytosolic expression pattern with a few puncta in well-fed animals (Fig 2AA and 2BB, white arrowheads). The characteristics of these makers change depending on the state of autophagic flux (*vide infra*).

The water channel, AQP-1::CemOrange2, localized to the intestinal plasma membrane (Fig 2CC and 2DD) with bright fluorescence evident at both the apical (Fig 2DD, white asterisk) and basolateral membranes (Fig 2DD, white arrowhead).

### Trafficking of CemOrange2 fusion proteins

The transgenic lines described above demonstrated subcellular distribution profiles consistent with those described in previously published reports. However, we sought an independent means to confirm that CemOrange2 markers trafficked to the correct positions by performing colocalization studies with a different set of previously reported genes, target peptides or fluorescent stains known to localize to specific organelles. Colocalization was assessed using the Manders Correlation Coefficient (MCC) with a value ≤ 0.5 showing no colocalization, 0.5–0.8 demonstrating partial colocalization and ≥ 0.8 corresponding to high colocalization [23].

**Fig 3:**
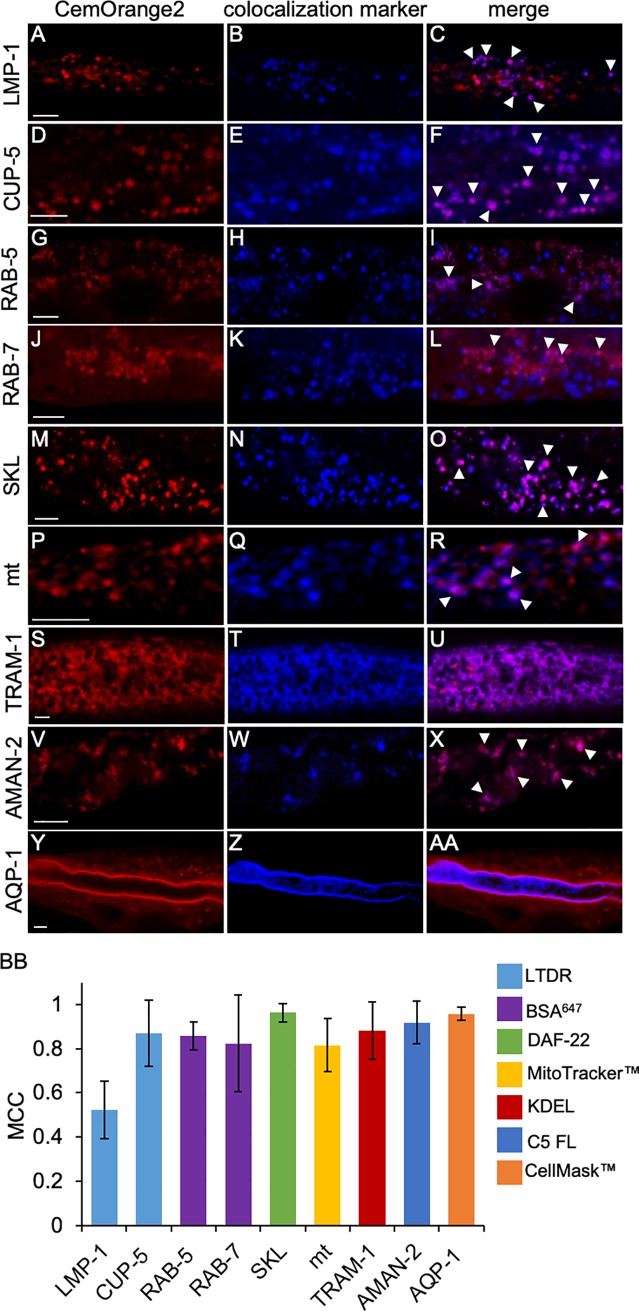
Colocalization of CemOrange2 probes with differentially labeled subcellular markers. Transgenic animals expressing the LMP-1, CUP-5, RAB-5, RAB-7, SKL, mt, TRAM-1, AMAN-2 or AQP-1 markers fused to CemOrange2 were labeled with previously described subcellular organelle markers (colocalization marker) and examined by confocal microscopy. Images were acquired over >20 *z*-planes using a 40x PlanApo oil immersion objective (N.A. 1.3) at wavelengths *Ex*555 nm/*Em*565-590 nm and a single XY-plane of representative images of animals are shown. To determine the subcellular localization, LMP-1::CemOrange2 (A; red) and CemOrange2::CUP-5 (D; red) expressing transgenic *C*. *elegans* were incubated with LTDR (B and E, blue) to label acidic compartments such as the lysosome. LMP-1 and CUP-5 colocalized with LTDR staining (C and F; magenta, arrowheads). Note, GLO-1 did not colocalize with LTDR (S1 Fig). CemOrange2::RAB-5 (G, red) and CemOrange2::RAB-7 (J, red) expressing animals were incubated with BSA::AlexaFluor^647^ (H and K, blue) to label the endo-lysosomal compartments. Both RAB-5 and RAB-7 colocalized with BSA::AlexaFluor^647^ (I and L, arrowheads). Transgenic animals expressing both CemOrange2::SKL (M, red) and DAF-22::GFP (N, blue) colocalized (O; magenta, arrowheads). ^mt^CemOrange2 (P, red) expressing *C*. *elegans* were incubated with MitoTracker Deep Red (Q, blue) to label mitochondria. ^mt^CemOrange2 colocalized with the MitoTracker stain (R; magenta, arrowheads). Transgenic animals expressing both CemOrange-1::TRAM-1 (S, red) and GFP::KDEL ER marker (T, blue) colocalized (U, magenta). AMAN-2::CemOrange2 (V, red) expressing animals were incubated with BODIPY C5 Ceramide-FL (C5 FL) (W, blue) to label lipid dense compartments such as the Golgi. AMAN-2 colocalized with C5 FL (X; magenta, arrowheads). AQP-1::CemOrange2 (Y) expressing animals were incubated in CellMask (Z; blue) to label phospholipid bilayers. AQP-1 colocalized with CellMask on the apical side of the plasma membrane (AA, magenta). Scale bar = 5 μm. (BB) Single XY plane confocal images of multiple transgenic *C*. *elegans* and their respective colocalization marker were analyzed for colocalization events (n ≥ 5). Colocalization was determined using the Manders correlation coefficient (MCC) algorithm in Volocity image analysis software (v6.3) analyzing the ratio of pixels in the CemOrange2 channel to pixels in the colocalization marker channel.

To confirm that LMP-1::CemOrange2 (Fig 3A) and CUP-5::CemOrange2 (Fig 3D) localized to lysosomes, transgenic animals expressing either fusion protein were labeled with LTDR, a lysosomotropic probe that fluoresces in acidic conditions (Figs 3B and 2E). Transgenic *C*. *elegans* expressing LMP-1:: CemOrange2 partially colocalized with LTDR staining (Fig 3C, merge, white arrowheads) with an average MCC = 0.52 (Fig 3BB). However, the majority of the CUP-5::CemOrange2 positive vesicles colocalized under the same staining conditions (Fig 3F, merge, white arrowheads) with an average MCC = 0.87 (Fig 3BB). The GLO-1::CemOrange2 positive vesicles in *C*. *elegans* did not colocalize with LTDR (S1A–S1C Fig) consistent with the protein localization to LRO’s rather than lysosomes. To further confirm these findings, plasmids containing P_*nhx-2*_*glo-1*::*CemOrange2* were injected into transgenic animals expressing either LMP-1::GFP under the *vha-6* intestinal specific promoter (a kind gift from Dr. B. Grant) or GLO-1::GFP under the *ges-1* intestinal specific promoter (VS17). Transgenic animals expressing LMP-1::GFP (S1D Fig) and GLO-1::CemOrange2 (S1E Fig) did not colocalize (S1F Fig) consistent with the LTDR data (S1A–S1C Fig). Transgenic animals expressing GLO-1::GFP (S1G Fig) and GLO-1::CemOrange2 (S1H Fig) colocalized (S1I Fig) suggesting that CemOrange2 did not affect the trafficking of the GLO-1 protein. Taken together, these data suggested that GLO-1::CemOrange2 did not localize to the lysosome and that CemOrange2 did not affect the trafficking of the GLO-1 protein.

Alexa Fluor 647conjugate (BSA^647^) is a high molecular weight fluorescent tracer taken up by endocytosis and traffics through early (RAB-5^+^) and then late (RAB-7^+^) endosomes to lysosomes (Fig 3H and 3K) [48]. Both CemOrange2::RAB-5 (Fig 3G) and CemOrange2::RAB-7 (Fig 3J) colocalized with BSA^647^ (Fig 3I and 3L, white arrowheads) with an average MCC = 0.86 and 0.82, respectively (Fig 3BB).

*daf-*22, a homologue to the human sterol carrier protein, along with GFP::SKL, localizes to the peroxisome in wild-type animals [49]. To determine if the CemOrange2 FP altered trafficking of the SKL motif, DAF-22::GFP transgenic *C*. *elegans* were injected with the P_*nhx-2*_*CemOrange2*::*SKL* plasmid. CemOrange2::SKL (Fig 3M) and GFP::DAF-22 (Fig 3N) colocalized (Fig 3O, white arrowheads) with an average MCC = 0.96 (Fig 3BB).

MitoTracker Deep Red is a fluorescent probe that accumulates within mitochondria. Transgenic animals expressing CemOrange2 with the N-terminal mitochondrial localization tag (^mt^CemOrange2, Fig 3P) were stained with MitoTracker Deep Red and examined by confocal microscopy. The ^mt^CemOrange2 protein colocalized with MitoTracker Deep Red (Fig 3R; white arrowheads) with an average MCC = 0.82 (Fig 3BB).

The ER retention signal, KDEL, fused to the C-terminus of secreted FPs is often used as an ER and Golgi localization marker. We generated transgenic *C*. *elegans* strains by injecting the plasmids P_*nhx-2*_*GFP*::*KDEL* (Fig 3T) and P_*nhx-2*_*CemOrange2*::*tram-1* (Fig 3S). CemOrange2::TRAM-1 colocalized with the GFP::KDEL (Fig 3U, yellow) with an average MCC = 0.88 (Fig 3BB).

Golgi stacks were labeled using C5 FL (Fig 3W). Almost all AMAN-2::CemOrange2 (Fig 3V) colocalized with C5 FL (Fig 3X, white arrowheads) with an average MCC = 0.92 (Fig 3BB).

The apical plasma membrane is labeled using CellMask, an amphipathic molecule that exhibits both lipophilic and hydrophilic moieties. AQP-1::CemOrange2 (Fig 3Y) and CellMask (Fig 3Z) colocalized (Fig 3AA, magenta) with an average MCC = 0.91 (Fig 3BB). Taken together, these data suggested that CemOrange2 did not affect the trafficking of fusion proteins or peptides described in this report.

### The biological application of CemOrange2 fusion proteins

**Fig 4:**
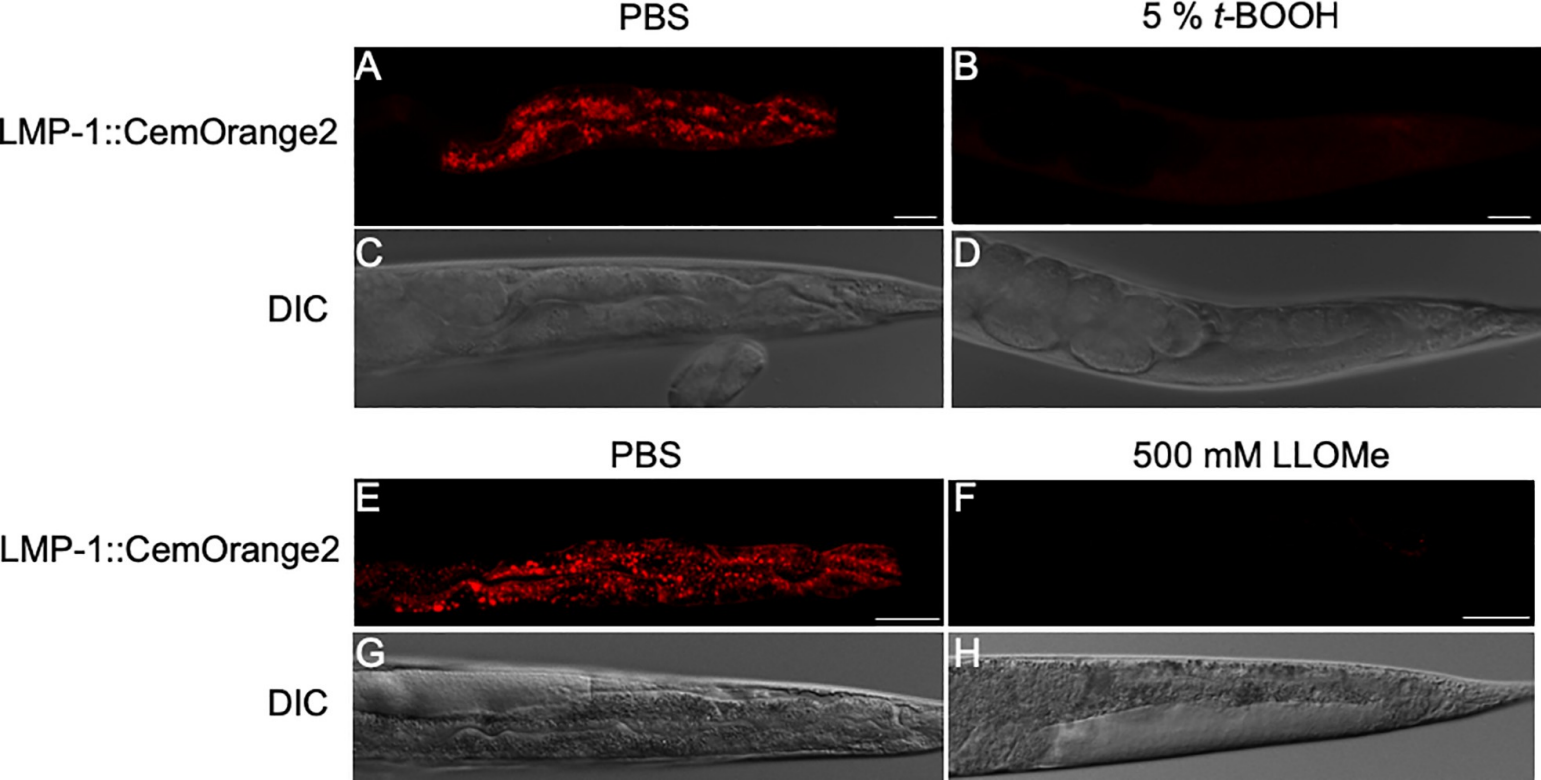
A live-animal sensor for lysosomal membrane integrity. P_*nhx-2*_*lmp-1*::*CemOrange2* transgenic *C*. *elegans* were exposed to either PBS (A, C, E, G) or 5% *tert*-butyl hydroperoxide (*t*-BOOH; B and D) in PBS for 30 min or 500 mM LLOMe (F and H) in PBS for 30 min. Animals were visualized by confocal microscopy using a 40x PlanApo oil immersion objective (N.A.1.3) at *Ex*555 nm/*Em*565-590 nm over >20 *z-*planes. Representative confocal images of animals shown as a single XY plane. Transgenic *C*. *elegans* exposed to PBS alone (A, C, E, G) have punctate LMP-1::CemOrange2 expression (A and E, red). The same animals exposed to 5% t-BOOH (B) or 500 mM LLoMe (F) lose punctate LMP-1::CemOrange2 labeled structures. DIC images (C, D, G, H) displayed for reference. Scale bars = 25 μm.

To assess the bioapplicability of the subcellular organelle markers, transgenic strains expressing a CemOrange2 fusion protein were exposed to different stressors. Lysosomal membrane permeabilization (LMP) is the hallmark of necrotic cell death in the *C*. *elegans* enterocyte [50, 51]. LMP was induced in wild-type animals expressing LMP-1::CemOrange2 by the ROS inducer, *tert*-Butyl hydroperoxide (*t-*BOOH) [50] or the lysosomotropic agent Leu-Leu-OMe (LLOMe) [52]. After exposure to 5% *t-*BOOH (Fig 4B and 4D) or 500 mM LLOMe (Fig 4F and 4H), the LMP-1::CemOrange2 staining lost the punctate staining pattern and became faintly diffuse cytosolic fluorescence (Fig 4B and 4F) as compared to the untreated controls (Fig 4A and 4E) suggesting a loss of lysosomal integrity. These studies indicated that LMP-1::CemOrange2 can be used to monitor lysosomal injury.

**Fig 5:**
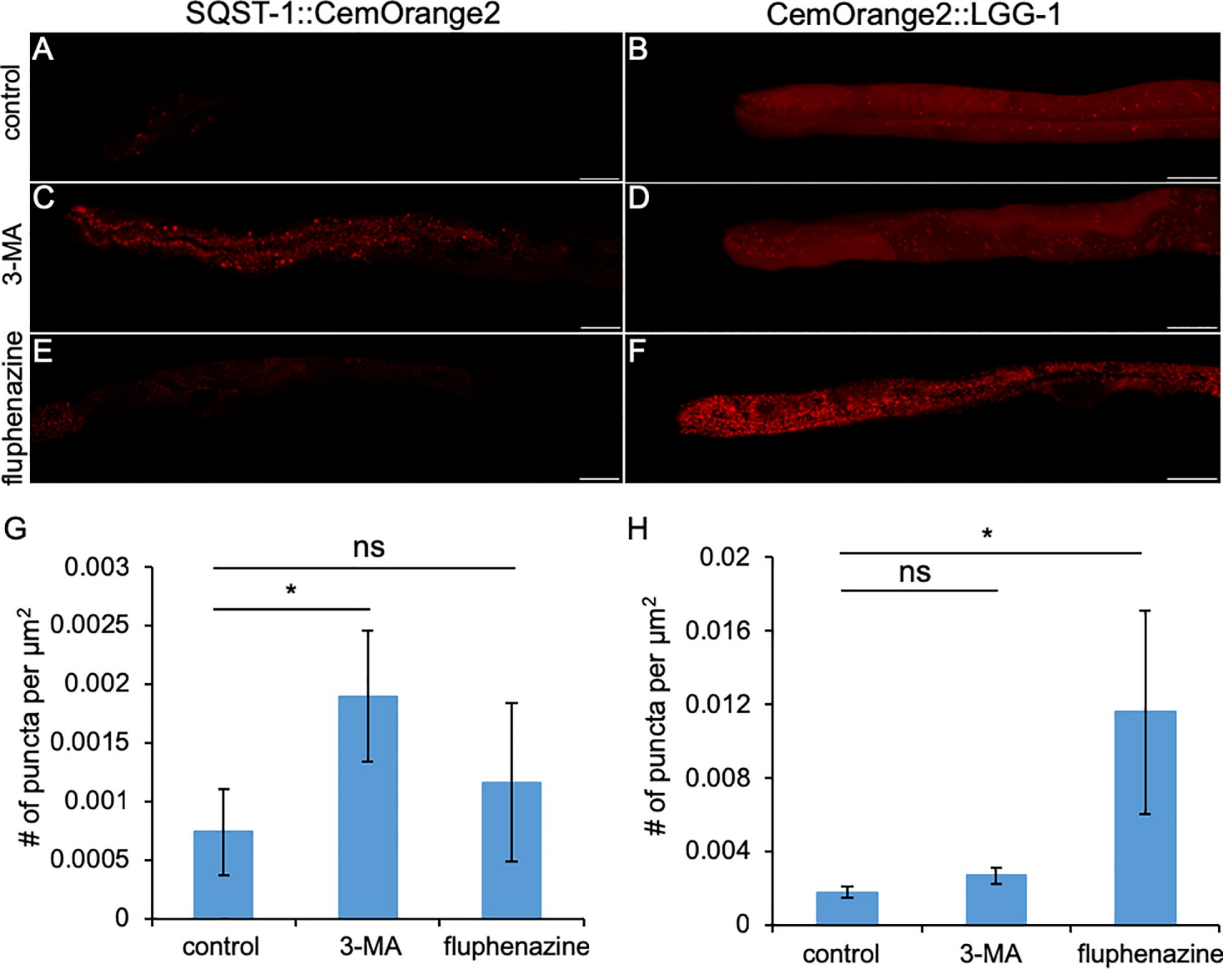
SQST-1:: and ::LGG-1 CemOrange2 fusion proteins monitor autophagic flux. P_*nhx-2*_*sqst-1*::*CemOrange2* (A, C, E; red) and P_*nhx-2*_*CemOrange2*::*lgg-1* (B, D, F; red) transgenic animals were treated with a diluent control (0.1% DMSO; A and B), 3-methyladenine (10 mM 3-MA, C and D), or 25 μM fluphenazine (E and F) for 16 hrs in liquid culture and analyzed using confocal microscopy over ≥ 20 *z*-planes *Ex*555 nm/*Em*565-590 nm. Representative confocal images of animals shown as a single XY plane. Scale bar = 25 μm. Multiple confocal images of transgenic animals (n ≥ 5 animals) expressing SQST-1::CemOrange2 (G) or CemOrange2::LGG-1 (H) after treatment with the above compounds, were quantified for number of puncta per μm^2^ imaged using the Quantification module in the Volocity image analysis software (v6.3). Statistical analysis of the drug-treated animals relative to diluent control was performed using an unpaired, 2-tailed *t*-test (*p<0.05).

During autophagy, SQST-1 (p62) binds ubiquitinated misfolded protein cargoes and recruits them to the developing autophagosome by binding LGG-1 (LC3/ATG8) [53]. To determine if these probes could be used to monitor autophagic flux, *C*. *elegans* strains were treated with either the autophagy enhancer, fluphenazine (25 μM) or the autophagy inhibitor, 3-methyladenine (3-MA, 10 mM) [32, 54]. *C*. *elegans* expressing SQST-1::CemOrange2 or CemOrange2::LGG-1 treated with the diluent, DMSO, served as a controls for baseline levels of autophagy in well-fed animals. As shown previously, SQST-1::CemOrange2 was barely detectable under baseline conditions (Fig 5A and 5G). As expected, CemOrange2::LGG-1 animals demonstrated a diffuse cytosolic pattern with a few punctate structures indicative of baseline autophagy (Fig 5B and 5H). Inhibition of autophagy by 3-MA, a type III phosphatidylinositol 3-kinase inhibitor that prevents autophagosome formation, blocked autophagic flux resulting in the accumulation of SQST-1::CemOrange2^+^ aggregates (Fig 5C and 5G). However, treatment with 3-MA had no detectable effect on baseline CemOrange2::LGG-1 distribution (Fig 5D and 5H). In contrast, treatment with fluphenazine significantly increased the number of CemOrange2::LGG-1^+^ punctate structures (autophagosomes) and had no detectable effect on SQST-1::CemOrange2 expression (Fig 5E, 5F, 5G and 5H). We concluded that autophagy markers fused to CemOrange2 function as expected under conditions that enhanced or inhibited autophagy.

### Three-color imaging

**Fig 6:**
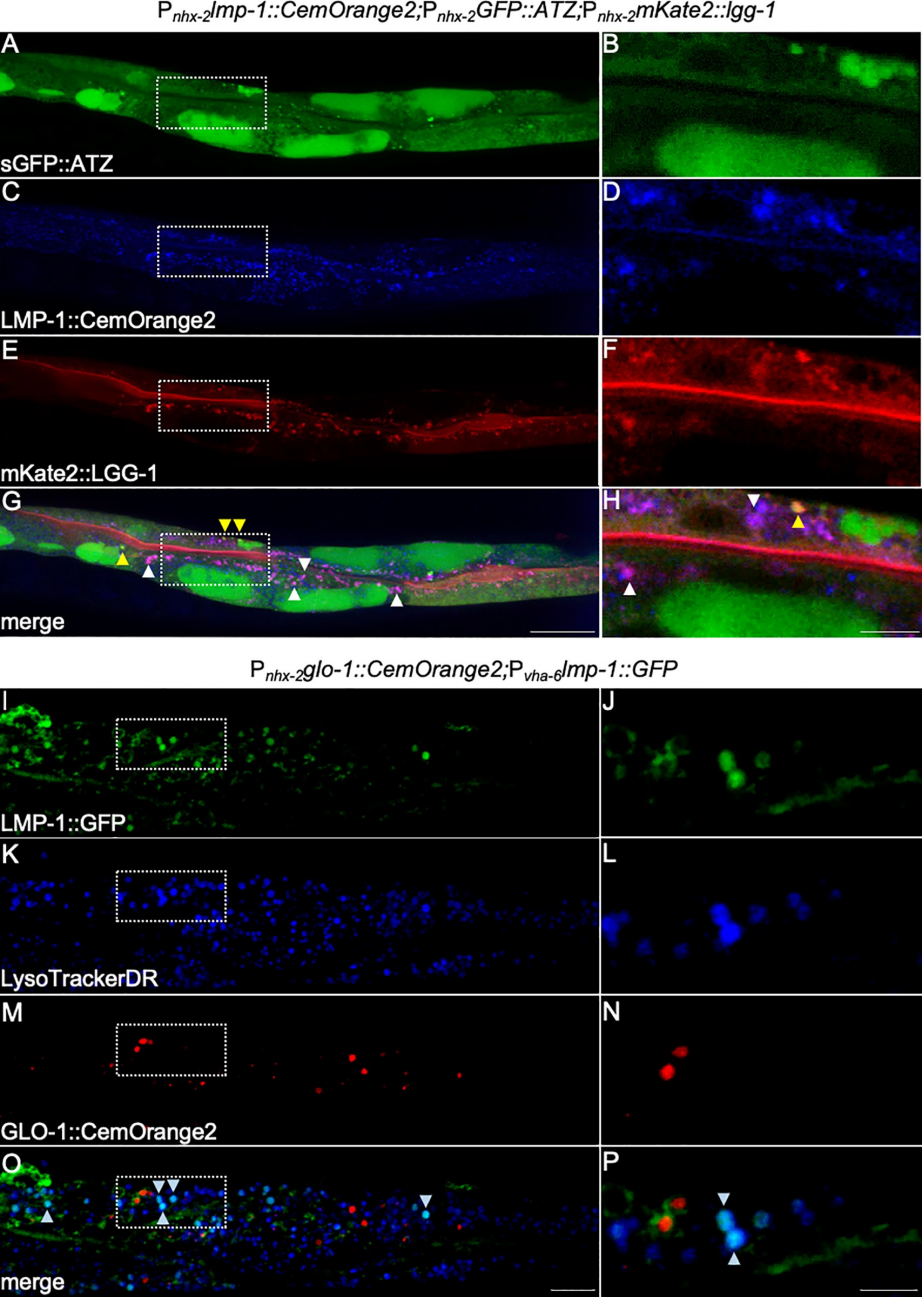
CemOrange2 and three-color imaging. P_*nhx-2*_*lmp-1*::*CemOrange2;*P_*nhx-2*_*GFP*::*ATZ;*P_*nhx-2*_*mKate2*::*lgg-1* transgenic *C*. *elegans* expressing sGFP::ATZ (A and B; green; *Ex*488 nm/*Em*500-540 nm), LMP-1::CemOrange2 (C and D; pseudocolored blue; *Ex*545 nm/ *Em*560-590 nm) and mKate2::LGG-1 (E and F; red; *Ex*594 nm/*E*m605-645 nm) were examined by confocal microscopy over >20 *z-*planes using a 40x PlanApo oil immersion objective (N.A. 1.3). Maximum intensity projections are shown (A, C, E, G). Scale bars = 25 μm. Magnified single XY regions (dashed box) are included to highlight colocalization events (B, D, F, H). Scale bars = 5 μm. mKate2::LGG-1 and LMP-1::CemOrange2 had multiple colocalization events (G and H; magenta merge, white arrowheads). Alpha-1 antitrypsin Z mutation (ATZ), a polymerizing protein which accumulates in the ER and is degraded, in part, by autophagy, show colocalization with mKate2::LGG-1 (G and H; orange merge, yellow arrowheads). P_*nhx-2*_*glo-1*::*CemOrange2;*P_*vha-6*_*lmp-1*::*GFP* transgenic *C*. *elegans* expressing LMP-1::GFP (I and J, green; *Ex*488 nm/*Em*500-540 nm) and GLO-1::CemOrange2 (M and N, red; *Ex*555 nm/*Em*560-590 nm) were stained with the acidic organelle fluorescent dye, LTDR (K and L, blue; *Ex*647 nm/*Em*660-700 nm). Animals were imaged by confocal microscopy over >20 *z-*planes and maximum intensity projections are shown (I, K, M, O). Scale bars = 25 μm. Magnified single XY regions (dashed box) are included to highlight colocalization events (J, L, N, P). Scale bars = 5 μm. Colocalization events are seen between LMP-1::GFP and LTDR (O and P, cyan merge, arrowheads) but not with GLO-1::CemOrange2.

To determine the utility of the CemOrange2 FP in multifluorophore imaging, we generated a transgenic line harboring three different *nhx-2*-driven transgenes expressing proteins fused to three different FPs; mKate2::LGG-1, LMP-1::CemOrange2, and sGFP::ATZ [27]. ATZ is a mutant form of the human serpin, μ1-antitrypsin/SERPINA1. The Z mutation causes ATZ to misfold in the ER and accumulate as polymers and aggregates [55, 56]. Using a modified CemOrange2 spectra in both the excitation and emission spectrum (*Ex*545 nm/ *Em*560-590 nm) and sequential scanning, the CemOrange2 FP was distinguished from both GFP and mKate2 (Fig 6A–6H). This combination of probes also confirmed several features related to the degradation of ATZ. Macroautophagy is a major degradation pathway for ER-retained sGFP::ATZ. At higher resolution, sGFP::ATZ was detected in LGG^+^ puncta (i.e., autophagosomes) as evident by the merge of GFP and mKate2 signals (Fig 6G and 6H, yellow arrowheads). The colocalization of autophagosomes with lysosomes is evident in the merge of mKate2 and CemOrange2 signals (Fig 6G and 6H; white arrowheads). The absence of GFP from these latter structures likely reflects the instability of GFP in acidic vesicles.

In another example, a transgenic *C*. *elegans* strain expressing both P_*vha-6*_LMP-1::GFP and P_*nhx-2*_GLO-1::CemOrange2 was stained with LTDR and imaged as described above (Fig 6I–6P). Representative images show there is minimal overlap between the fluorophores. As expected, there was extensive overlap between LTDR^+^ vesicles merged with GFP^+^ lysosomes (Fig 6O and 6P; arrowheads). Taken together, these data demonstrated that CemOrange2 was useful for live-animal multifluorophore imaging.

### Pathological variant validation by using co-localization markers

**Fig 7:**
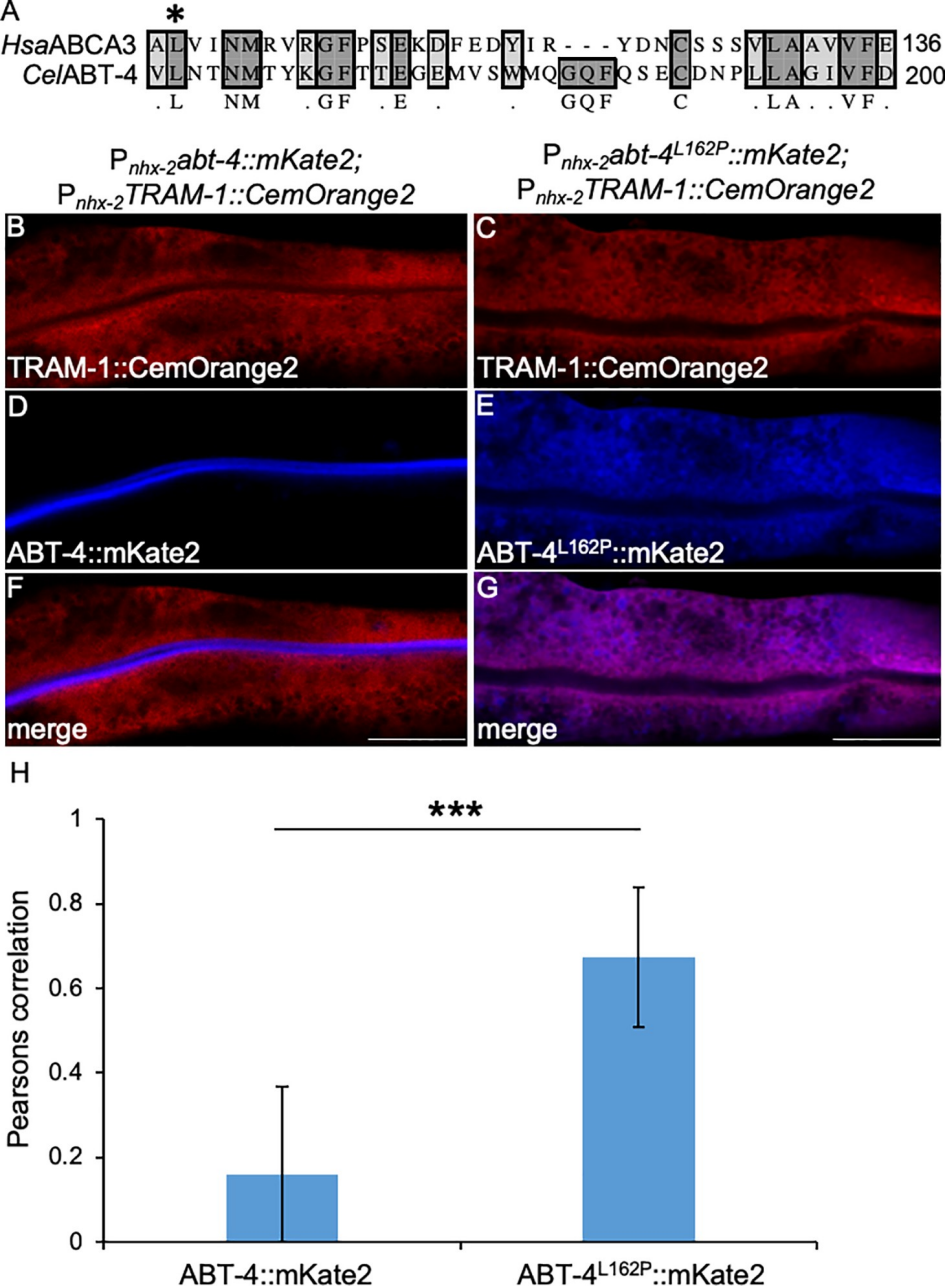
A CemOrange2 marker co-localizes a mutant ABC transporter protein to the ER. Alignment of the primary amino sequence from human (*Hsa*) ABCA3 (Accession: AAH20724.1) and *C*. *elegans* (*Cel*) ABT-4 (Accession: NP_503175.1) using the ClustalW algorithm (A). Only residues 100–136 of *Hsa*ABCA3 aligned with residues 161–200 from *Cel*ABT-4 are shown. The asterisk marks L101 (human numbering) or L162 (*C*. *elegans* numbering) in the alignment that is conserved between species. P_*nhx-2*_*abt-4*::*mKate2;*P_*nhx-2*_*TRAM-1*::*CemOrange2* (B, D, F) or P_*nhx-2*_*abt-4*^*L162P*^::*mKate2;*P_*nhx-2*_*TRAM-1*::*CemOrange2* (C, E, G) transgenic *C*. *elegans* strains were imaged by confocal microscopy using a 40x PlanApo oil immersion objective (N.A. 1.3) over >20 *z-*planes. Representative single XY regions are shown. ABT-4::mKate2 (D, pseudocolored blue) or ABT-4^L162P^::mKate2 (E, pseudocolored blue; *Ex* 594 nm/*Em*605-645 nm) and TRAM-1::CemOrange2 (B,C, orange; *Ex*549 nm/ *Em*560-580 nm), showed that ABT-4::mKate2 trafficked normally to the apical membrane in intestinal cells (F), whereas the single point mutation in ABT-4^L162P^ triggered retention within the ER (G). Scale bar = 25 μm. (H) Colocalization between ABT-4 or ABT-4^L162P^ and TRAM-1 (F and G, respectively) was determined using the Pearson correlation in Volocity image analysis software (v6.3) (***p<0.001; n≥10).

Model organisms, such as *C*. *elegans*, *D*. *rerio* and *D*. *melanogaster*, have proven to be essential systems in determining whether human variants of unknown significance (VUS) are pathologic [10, 11]. In *C*. *elegans*, genotype-pathologic phenotype correlations can be determined by comparing wild-type versus mutant human transgenes or by generating homologous mutations in orthologous genes. One means to assess these phenotypes is by using microscopic analysis to determine whether the variant of interest perturbs cellular dynamics or by altering the proteins subcellular distribution [2, 3]. The generation of the CemOrange2 organelle marker toolbox described here will be useful in identifying the subcellular phenotypic changes associated with a pathological VUS. As an example, human ABCA3 is a phospholipid transporter required for assembly of pulmonary surfactant in lamellar bodies and lamellar body biogenesis in type 2 pulmonary alveolar epithelial cells [57]. Different types of ABCA3 mutations disrupt surfactant synthesis and cause neonatal respiratory failure or childhood interstitial lung disease (chILD) in older infants and children [57–61]. One mutation, L101P, results in protein misfolding and accumulation within the ER. To determine if this pathologic variant can be detected in *C*. *elegans*, a mutated transgene (the conserved leucine is at position 162 in *C*. *elegans*) containing the worm orthologue of ABCA3, *abt-4*, fused to the N-terminus of mKate2 was introduced into wild-type animals (Fig 7A, asterix). Wild-type ABT-4::mKate2 was trafficked to the intestinal cell membrane (Fig 7B, 7D and 7F) however, similar to that observed in mammalian cell lines, ABT-4^L162P^ was retained in the ER of intestinal cells of *C*. *elegans* (Fig 7C, 7E, 7G and 7H). These results confirm that *C*. *elegans* can be used to assess human VUSs.

## Discussion

The purpose of this study was to generate a panel of FP markers for subcellular structures that could be used for multiplex imaging in *C*. *elegans*, one of the premier model organisms for studying cell biological and developmental processes in real-time [2, 3, 11]. The challenge was to select a FP that did not aggregate spontaneously *in vivo*, and displayed an *Ex/Em* spectra that was distinct from the popular FPs already adapted for use in *C*. *elegans* (e.g., GFP, YFP, CFP and mCherry) [1, 5, 7–9]. The ideal fluorescent protein should also have a fast maturation time, high brightness and high photostability. Of the fluorescent proteins that we chose to study, mCardinal has the highest photostability and a fast maturation time (Table 1). However, it possesses approximately half the brightness of EGFP (Table 1). While the maturation time of mNeptune2.5 is similar to EGFP, it has approximately 70% the brightness and the photostability is unknown. Additionally, the reported *Ex*_*max*_/*Em*_*max*_ of mNeptune2.5 is closest to both mCherry and mKate2, which would make it more difficult to spectrally separate from these 2 commonly used fluorophores. While mOrange2 has a reported slow maturation time of 270 minutes, it has a brightness similar to EGFP and has a high photostablity of 228 seconds (Table 1). After codon adaptation, we found that CemOrange2, had an *Ex*_*max*_*/Em*_*max*_ spectra *in vivo* that placed it between those of YFP and mKate2, and that the signal from this FP was easily distinguished from *C*. *elegans* autofluorescence (S2 Fig). While CemOrange2 could be spectrally distinguished from the far red mKate2 *in vivo*, this FP spectra would overlap with other commonly used red FPs, such as DsRed (*Ex*_*max*_/*Em*_*max*_ 558/553) and dTomato (*Ex*_*max*_/*Em*_*max*_ 554/581). In contrast, both the *in vivo* CemNeptune2.5 and CemCardinal emission and excitation spectra overlapped significantly with mKate2, which would confound colocalization studies. Moreover, the CemOrange2 FP demonstrated a diffuse and homogenous cytoplasmic distribution when expressed in *C*. *elegans* intestinal cells. In contrast, CemCardinal2, a monomeric FP, showed both a diffuse and granular distribution pattern. We did not determine whether CemCardinal2 formed aggregates *in vivo*, or associated with a vesicular structure due to a unique aspect of its structure. Regardless, we were reluctant to utilize CemCardinal2 as this characteristic could confound its use in colocalization studies. Another advantage of CemOrange2 is that it also avoids the phototoxicity associated with the distinct *Ex/Em* spectra of BFPs [62]. While the confocal setup described here uses a supercontinuum white light laser source, more traditional confocal and widefield systems can be used to to visualize CemOrange2. Indeed, CemOrange2 was detectable using a 561nm wavelength excitation laser (S3D Fig). Additionally, there was no observable cross talk with spectral channels visualizing the GFP or mKate2 fluorophore excitation and emission settings (*Ex488* nm/*Em500-540* nm and *Ex594* nm/*Em605-640* nm, respectively; S3A–S3F Fig). Moreover, tuning the white light laser source to a more conventional single line laser imaging system using excitation wavelengths of 488nm, 561nm and 594nm, we were still able to separate GFP, CemOrange2 and mKate2 FPs in the P_*nhx-2*_*lmp-1*::*CemOrange2;*P_*nhx-2*_*GFP*::*ATZ;*P_*nhx-2*_*mKate2*::*lgg-1* transgenic *C*. *elegans* (S3G–S3L Fig).

We generated a series of plasmids containing a CemOrange2 minigene fused to a targeting sequence or another gene expressing a protein known to target to a unique subcellular organelle or location. Using confocal microscopy, the subcellular distribution patterns of these markers suggested that they were targeted to the correct location. However, FPs can affect the folding and conformation of their fusion partner so we sought an independent means to assure that the CemOrange2 markers were targeting the correct cellular address [63]. In all nine cases tested, the CemOrange2 fusions showed a significant colocalization with a non-overlapping fluorophore targeting the same structure (MCC>0.5). Taken together, these studies showed that CemOrange2 FPs were directed to the correct subcellular addresses and should prove useful for multicolor live-cell imaging. Indeed, this functionality was demonstrated by a transgenic strain expressing human alpha-1 antitrypsin with the Z mutation fused to the C-terminus of GFP. This aggregation-prone protein, is retained in the ER and is partially degraded by macroautophagy [27, 28, 32, 56]. Examination of these animals showed colocalization of ATZ in LGG-1^+^ (a *C*. *elegans* orthologue of LC3) structures (i.e., autophagosomes). The identification in of autophagosomes with a specific cargo is difficult in real-time and underscores the sensitivity of multifluorophore imaging technology.

Knowledge about human variants of unknown significance can be obtained by determining whether the phenotype of the variant differs from the wild-type gene when expressed in model organisms, such a *C*. *elegans*, *D*. *rerio*, *D*. *melanogaster* or *S*. *cerevisiae* [2, 3, 10, 11]. Some abnormal phenotypes are straightforward in their presentation at the whole organism level, if they result in, for example, abnormal development of decreased longevity. However, many abnormal phenotypes are subtler in their presentation and are manifest only after the application of a cellular stress or by examining subcellular functions; for example, protein misfolding disorders. Many of these proteostasis disorders only manifest after the aggregation-prone or misfolded proteins accumulate over time. Classical examples are neurodegenerative disorders associated with the aberrant accumulation of Huntingtin, Aß, alpha-synuclein and/or tau [64–67]. In many cases, alterations in the subcellular distribution of these proteins are detected long before a pathologic phenotype emerges. For this reason, multifluorophore imaging in *C*. *elegans* can be a useful adjunct to the analysis of VUS, especially those associated with the subtle effects associated with protein misfolding. We showed that a mutation in the *C*. *elegans* ABC transporter, *abt-4*, resulted in ER retention, which is exactly what occurs in the human orthologous gene, ABCA3, with an identical mutation at a single conserved amino acid [57–61]. Similar results were observed in *C*. *elegans* expressing the human pathologic variant Z of alpha-1 antitrypsin [27, 28, 32]. Taken together, these studies show that the addition of CemOrange2 to the *C*. *elegans* FP toolbox expands their ability to assess human VUS behavior by multifluorophore, real-time subcellular imaging.

## Supporting information

S1 Table PCR primer pairs used for transgene construction. (DOCX)

S2 Table *C. elegans* strains used in this study. (DOCX)

S1 Fig The lysosomal related organelle marker, GLO-1, does not colocalize with lysosomes. A-C) Transgenic *C*. *elegans* expressing GLO-1:: CemOrange2 (A; red, *Ex*549 nm/*Em*560-585 nm) were stained with LTDR (B, blue; *Ex*647 nm/*Em*660-700 nm) and examined by confocal microscopy over >20 *z*-planes using a 40x PlanApo oil immersion objective (N.A. 1.3). Note the lack of colocalization as shown by discrete blue and red puncta (C, merge). The MCC of this representative image was 0.18, indicating the absence of colocalization. P_*nhx-2*_*glo-1*::*CemOrange2;*P_*vha-6*_*lmp-1*::*GFP* (D and E) transgenic *C*. *elegans* were imaged by confocal microscopy over >20 *z-*planes using a 63x PlanApo oil immersion objective (N.A. 1.4). LMP-1::GFP puncta (D and F, green; *Ex*488 nm/*Em*500-540 nm) do not colocalize with the GLO-1::CemOrange2 (E and F; red; *Ex*555 nm/*Em*565-590 nm). (G-I) CemOrange2 did not affect the trafficking of GLO-1. P_*nhx-2*_*glo-1*::*CemOrange2;*P_*ges-1*_*glo-1*::*GFP* (G and H) transgenic animals showed GLO-1::GFP positive (G, green; *Ex*488 nm/*Em*500-540 nm) and GLO-1::CemOrange2 puncta (H, red; *Ex*555 nm/*Em*565-590 nm) colocalized (I, merge yellow). Scale bars = 5 μm.
(TIFF)

S2 Fig CemOrange2 fluorescence is distinct from background autofluorescence in wild-type C. elegans. Either transgenic (A) P_*nhx-2*_*CemOrange2* or (B) wild-type (N2) *C*. *elegans* posterior intestines were imaged using a confocal microscope fitted with a white light laser and spectral detectors at varying excitation (range 470 nm-670 nm in 5 nm steps) and emission wavelengths (range 485–785 in 5 nm steps and a detection window of 20 nm). The fluorescence intensity (0–255) of each 8 bit image at each excitation and emission wavelength was plotted using the 2D bilinear excitation and emission lambda scan algorithm in the LASX software (Leica Microsystems, Buffalo Grove, IL) using the color gradient of fluorescence intensity indicated. The inset in B shows the autofluoresence of the wild-type *C*. *elegans* with a the look up table (LUT) rescaled to between 0 and 20 to show that autofluoresence is exhibited in the blue light range with minimal overlap with the CemOrange2 fluorescence spectrum.
(TIFF)

S3 Fig CemOrange2 can be visualized using standard excitation laser lines. Either P_*nhx-2*_*lmp-1*::*CemOrange2* (A-F) or P_*nhx-2*_*lmp-1*::*CemOrange2;*P_*nhx-2*_*GFP*::*ATZ;*P_*nhx-2*_*mKate2*::*lgg-1* (G-L) transgenic C. elegans were imaged using a confocal microscope fitted with a white light laser set at either optimized 488, 545 and 594 nm (A, C, E, G, I, K) or standard 488, 561 and 594 nm (B, D, F, H, J, L) excitation wavelengths in sequential imaging mode over ≥30 *z*-planes. At either optimized or standard imaging excitation wavelengths, P_*nhx-2*_*lmp-1*::*CemOrange2* was detected only with the 545 nm (C; blue) or 561 nm (D; blue) excitation laser settings with similar punctate distribution with no cross talk with the 488 nm (A, B; green) and 594 nm (E, F; red) excitation laser lines. P_*nhx-2*_*lmp-1*::*CemOrange2;*P_*nhx-2*_*GFP*::*ATZ;*P_*nhx-2*_*mKate2*::*lgg-1* transgenic *C*. *elegans* imaged under the same conditions showed that all three fluorophores were readily detected using optimized or standard imaging settings; GFP::ATZ (G, H; green) using the *Ex*488 nm laser line, LMP-1::CemOrange2 (I, J; blue) with either *Ex*545 nm (I) and *Ex*561 nm (J) laser line and mKate2::LGG-1 (K, L; red) with the *Ex*594 nm laser line indicating that CemOrange2 can be utilized with more standard confocal imaging systems. Scale bar = 25 nm.
(TIFF)
