# Supplementary material for: CemOrange2 fusions facilitate multifluorophore subcellular imaging in C. elegans
Source: PLoS One. 2019 Mar 26;14(3):e0214257. doi: 10.1371/journal.pone.0214257 (PMC6435234; doi:10.1371/journal.pone.0214257)
Supplement: S1 Table — (DOCX) [file pone.0214257.s001.docx]

**S1 Table. PCR primer pairs used for transgene construction**

| **primer set** | **forward**  **/reverse** | **sequence (5’-3’)** | **amplicon/insertion size (bp)** |
| --- | --- | --- | --- |
| 1 | F | ATATATAAGCTTGATTCATACTGAAGTAGGTGCC | 2033 |
|  | R | ATATATTCTAGACGGAGAAGAGACATGATTTAATCAC |  |
| 2 | F | ATTTTCAGGAGGACCCTTGGCTAGCAAAAATGGCTGCTGCATCATCC | 2439 |
|  | R | ATGGTACCAGGCCTCTCGAGGTGAAGAAGCGCCTGAAG |  |
| 3 | F | ATTTTCAGGAGGACCCTTGGCTAGCAAAAATGACGGCCGAGGAAGATAC | 1101 |
|  | R | TGGTACCAGGCCTCTCGAGAGCTTGAAGCAATTTTTGTTG |  |
| 4 | F | GTCCTAGGAGATCTGAATTCAAGTGGGCTTACAAGGAG | 425 |
|  | R | GGCCGATGCGGAGCTCTTAGGCGCCTTATTCCTTCTTTTCGACCTC |  |
| 5 | F | GCCACCCGTACCCTCTGCTCCTCCCGTACCCTCCTCGTCTCCAAGGGAGAGGAG | 75 |
|  | R | TGGCTTGAAGAAACGGATGGATTGACGGAGGGAGAGCATTTTTCCGCCCATGGTA |  |
| 6 | F | GAAGCGTAAGGTCGGCGGAAAAATGGTCTCC | 27 |
|  | R | TTCTTTGGGGCCATCATGGTACCAGGCCTCTC |  |
| 7 | F | AAGCGTAAGGTCGTCTCCAAGGGAGAGGAG | 75 |
|  | R | CTTCTTTGGGGCCATTTTTCCGCCCATGGTA |  |
| 8 | F | AACGGTCCTAGGAGATCTGAATTCTCTCGCCGGAGCACAACTG | 6889 |
|  | R | ACAGCGGCCGATGCGGAGCTCTTAGGCGCCTTATTTGAATAAATTACAATTTATTAAGTTCTCAAGAAATC |  |
| 9 | F | ATTTTCAGGAGGACCCTTGGAAAAATGTCATCTCGTAAAGG | 2129 |
|  | R | TGGTACCAGGCCTCTCGAGGCTAGCCATGATGGAACAACGATC |  |
| 10 | F | GCTCTAAGAGCTCCGCATCGGC | 9 |
|  | R | TTGGAGGCGCCGAATTCAGATCTC |  |
| 11 | F | CAGGAGGACCCTTGGCTAGCAAAAATGTTGAAATCGTTTGTCATC | 920 |
|  | R | ACCAGGCCTCTCGAGGACGCTGGCATATCCTTG |  |
| 12 | F | AACGGTCCTAGGAGATCTGAATTCGGCCGCCCGAAACGCAGGA | 1327 |
|  | R | ACAGCGGCCGATGCGGAGCTCTTAGGCGCCTTATTTACAGCATGAACCCTTTTGTTGCTGTTG |  |
| 13 | F | TAGGAGATCTGAATTCGGCGCCTCGGGAACCAGAAAGAAGGC | 1246 |
|  | R | CGATGCGGAGCTCTTATTAACAATTGCATCCCGAATTC |  |
| 14 | F | TAGGAGATCTGAATTCGGCGCCGGAGGAGGAGGATCCGTTAAGCCGCAAGGAGGG | 1471 |
|  | R | gatgcggagctcttagttaatttttcttcttcgaatcgctc |  |
| 15 | F | CAGGAGGACCCTTGGCTAGCAAAAATGGGAAAACGCAATTTC | 5397 |
|  | R | ACCAGGCCTCTCGAGAAATGATACAAGAATACTGGAAATATC |  |
| 16 | F | CAGGAGGACCCTTGGCTAGCAAAAATGGCAGCACTCACAAATAAC | 9298 |
|  | R | ACCAGGCCTCTCGAGGCAACATTTCGAGTCGTATC |  |
| 17 | F | TAGGAGATCTGAATTCGGCGCCGGAGGAGGAGGATCCGAACCGACAGCCGCCGAG | 1043 |
|  | R | CGATGCGGAGCTCTTATCATTGGGCCTTCTGAGCAG |  |
| 18 | F | GCATGGATGAACTATACAAAGACGAGCTCTAGCATTCGTAGAATTCCAAC | 12 |
|  | R | GTTGGAATTCTACGAATGCTAGAGCTCGTCTTTGTATAGTTCATCCATGC |  |
| 19 | F | AGAACATTTTCAGGAGGACCCTTGGAAAATGGGTGCATTGGG | 6813 |
|  | R | GTTCTCCTTAATCAGCTCGCTCACCATGCTAGCGAAATACATGGCACTTTCCGcg |  |
| 20 | F | AGTACCAGTTCCAAACACGAATATG | 0 |
|  | R | GCTGTGATATTGTAGAGTC |  |
