# Supplementary material for: CemOrange2 fusions facilitate multifluorophore subcellular imaging in C. elegans
Source: PLoS One. 2019 Mar 26;14(3):e0214257. doi: 10.1371/journal.pone.0214257 (PMC6435234; doi:10.1371/journal.pone.0214257)
Supplement: S2 Table — (DOCX) [file pone.0214257.s002.docx]

**S2 Table. *C. elegans* strains used in this study**

| **strain name** | **genotype** | **protein expressed** | **Figure(s)** |
| --- | --- | --- | --- |
| VK2620 | *vkEx2620[*P*_nhx-2_aman-2::CemOrange2;*P*_myo-2_GFP]* | AMAN-2::CemOrange2 | 2 and 3 |
| VK2664 | *vkEx2664[*P*_nhx-2_CemOrange2::tram-1;*P*_myo-2_GFP]* | CemOrange2::TRAM-1 | 2 |
| VK2666 | *vkEx2666[*P*_nhx-2_CemOrange2::rab-7;*P*_myo-2_GFP]* | CemOrange2::RAB-7 | 2 and 3 |
| VK2671 | *vkEx2671[*P*_nhx-2_CemOrange2::rab-5;*P*_myo-2_GFP]* | CemOrange2::RAB-5 | 2 and 3 |
| VK2674 | *vkEx2674[*P*_nhx-2_CemOrange2::pisy-1;*P*_myo-2_GFP]* | CemOrange2::PISY-1 | 2 |
| VK2688 | *vkEx2688[*P*_nhx-2_CemOrange2::cup-5;* P*_myo-2_GFP]* | CemOrange2::CUP-5 | 2 and 3 |
| VK2697 | *vkIs2697[*P*_nhx-2_lmp-1::CemOrange2;*P*_myo-2_GFP]* | LMP-1::CemOrange2 | 2, 3 and 4 |
| VK2700 | *vkEx2700[*P*_nhx-2_CemOrange2::SKL;*P*_myo-2_GFP]* ***** | CemOrange2::SKL | 2 |
| VK2702 | *vkEx2702[*P*_nhx-2_^mt^CemOrange2;*P*_myo-2_GFP]* **^#^** | ^mt^CemOrange2 | 2 and 3 |
| VK2728 | *vkEx2728[*P*_nhx-2_sqst-1::CemOrange2;*P*_myo-2_GFP]* | SQST-1::CemOrange2 | 2 |
| VK2733 | *vkEx2733[*P*_nhx-2_NLS^SV-40^::CemOrange2::NLS^egl-13^;*P*_myo-2_GFP]* **^†^** | NLS^SV-40^::CemOrange2:: NLS^egl-13^ | 2 |
| VK2734 | *vkIs2734[*P*_nhx-2_lmn-1::CemOrange2;*P*_myo-2_GFP]* | LMN-1::CemOrange2 | 2 |
| VK2735 | *vkEx2735[*P*_nhx-2_glo-1::CemOrange2;*P*_myo-2_GFP]* | GLO-1::CemOrange2 | 2 and S1A-C |
| VK2738 | *vkEx2738[*P*_nhx-2_CemOrange2::lgg-1;*P*_myo-2_GFP]* | CemOrange2::LGG-1 | 2 |
| VK2748 | *vkEx2748[*P*_nhx-2_CemOrange2::tram-1;*P*_nhx-2_GFP::KDEL]* | CemOrange2::TRAM-1; GFP::KDEL | 3 |
| VK2749 | *vkIs2749[*P*_nhx-2_lmp-1::CemOrange2;*P*_nhx-2_GFP::ATZ;*P*_nhx-2_mKate2::lgg-1*;P*_myo-2_GFP*;P*_myo-2_mCherry]* | LMP-1::CemOrange2; GFP::ATZ; mKate2::lgg-1 | 6 |
| VK2755 | *vkEx2755[*P*_nhx-2_CemOrange2;*P*_myo-2_GFP]* | CemOrange2 | 1 |
| VK2756 | *vkEx2756[*P*_nhx-2_CemCardinal2;*P*_myo-2_GFP]* | CemCardinal2 | 1 |
| VK2757 | *vkEx2757[*P*_nhx-2_CemNeptune2.5;*P*_myo-2_GFP]* | CemNeptune2.5 | 1 |
| VK2838 | *vkEx2838[*P*_nhx-2_CemOrange2::SKL;P_vha-6_GFP::DAF-22;*P*_myo-2_GFP]* | CemOrange2::SKL; GFP::DAF-22 | 3 |
| VK2877 | *vkIs2877[*P*_nhx-2_sqst-1::CemOrange2;*P*_myo-2_GFP]* | SQST-1::CemOrange2 | 5 |
| VK2878 | *vkIs2878[*P*_nhx-2_CemOrange2::lgg-1;*P*_myo-2_GFP]* | CemOrange2::LGG-1 | 5 |
| VK2881 | *vkIs2881[*P*_nhx-2_glo-1::CemOrange2;*P*_ges-1_glo-1::GFP;*P*_myo-2_GFP]* | GLO-1::CemOrange2; GLO-1::GFP | S1G-I |
| VK2882 | *vkIs2882[*P*_nhx-2_glo-1::CemOrange2;*P*_vha-6_lmp-1::GFP;*P*_myo-2_GFP]* | GLO-1::CemOrange2; LMP-1::GFP | 6 and S1D-F |
| VK2883 | *vkEx2883[*P*_nhx-2_aqp-1::CemOrange2;*P*_myo-2_GFP]* | AQP-1::CemOrange2 | 2 and 3 |
| VK3160 | *vkEX3160[*P*_nhx-2_abt-4mKate2;*P*_nhx-2_CemOrange2::tram-1]* | ABT-4::mKate2; CemOrange2::TRAM-1 | 7 |
| VK3161 | *vkEX3161[*P*_nhx-2_abt-4^L162P^mKate2;*P*_nhx-2_CemOrange2::tram-1]* | ABT-4^L162P^::mKate2; CemOrange2::TRAM-1 | 7 |

*Peroxisome target sequence = SKL

**^#^**mt (mitochondria target sequence) = MLSLRQSIRFFKPATRTLCSSRTLL

**^†^**NLS^SV-40^ = MAPKKKRKV; NLS^egl-13^ = MSRRRKANPTKLSENAKKLAKEVEN
