# Supplementary figures and images for: CemOrange2 fusions facilitate multifluorophore subcellular imaging in C. elegans
Source: PLoS One. 2019 Mar 26;14(3):e0214257. doi: 10.1371/journal.pone.0214257 (PMC6435234; doi:10.1371/journal.pone.0214257)

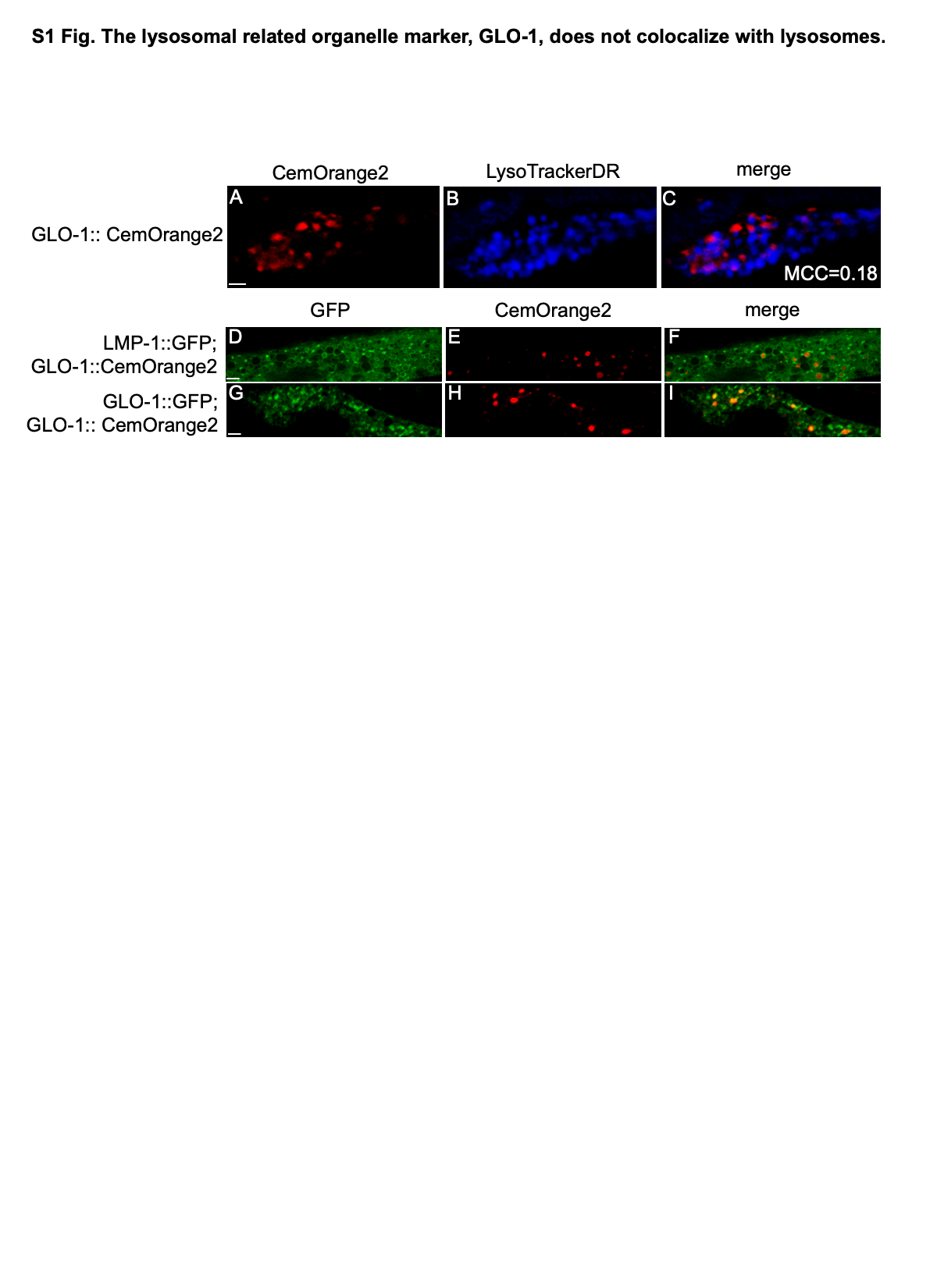

Supplement: S1 Fig — A-C) Transgenic C. elegans expressing GLO-1:: CemOrange2 (A; red, Ex549 nm/Em560-585 nm) were stained with LTDR (B, blue; Ex647 nm/Em660-700 nm) and examined by confocal microscopy over >20 z-planes using a 40x PlanApo oil immersion objective (N.A. 1.3). Note the lack of colocalization as shown by discrete blue and red puncta (C, merge). The MCC of this representative image was 0.18, indicating the absence of colocalization. Pnhx-2glo-1::CemOrange2;Pvha-6lmp-1::GFP (D and E) transgenic C. elegans were imaged by confocal microscopy over >20 z-planes using a 63x PlanApo oil immersion objective (N.A. 1.4). LMP-1::GFP puncta (D and F, green; Ex488 nm/Em500-540 nm) do not colocalize with the GLO-1::CemOrange2 (E and F; red; Ex555 nm/Em565-590 nm). (G-I) CemOrange2 did not affect the trafficking of GLO-1. Pnhx-2glo-1::CemOrange2;Pges-1glo-1::GFP (G and H) transgenic animals showed GLO-1::GFP positive (G, green; Ex488 nm/Em500-540 nm) and GLO-1::CemOrange2 puncta (H, red; Ex555 nm/Em565-590 nm) colocalized (I, merge yellow). Scale bars = 5 μm. (TIFF) [file pone.0214257.s003.tiff]

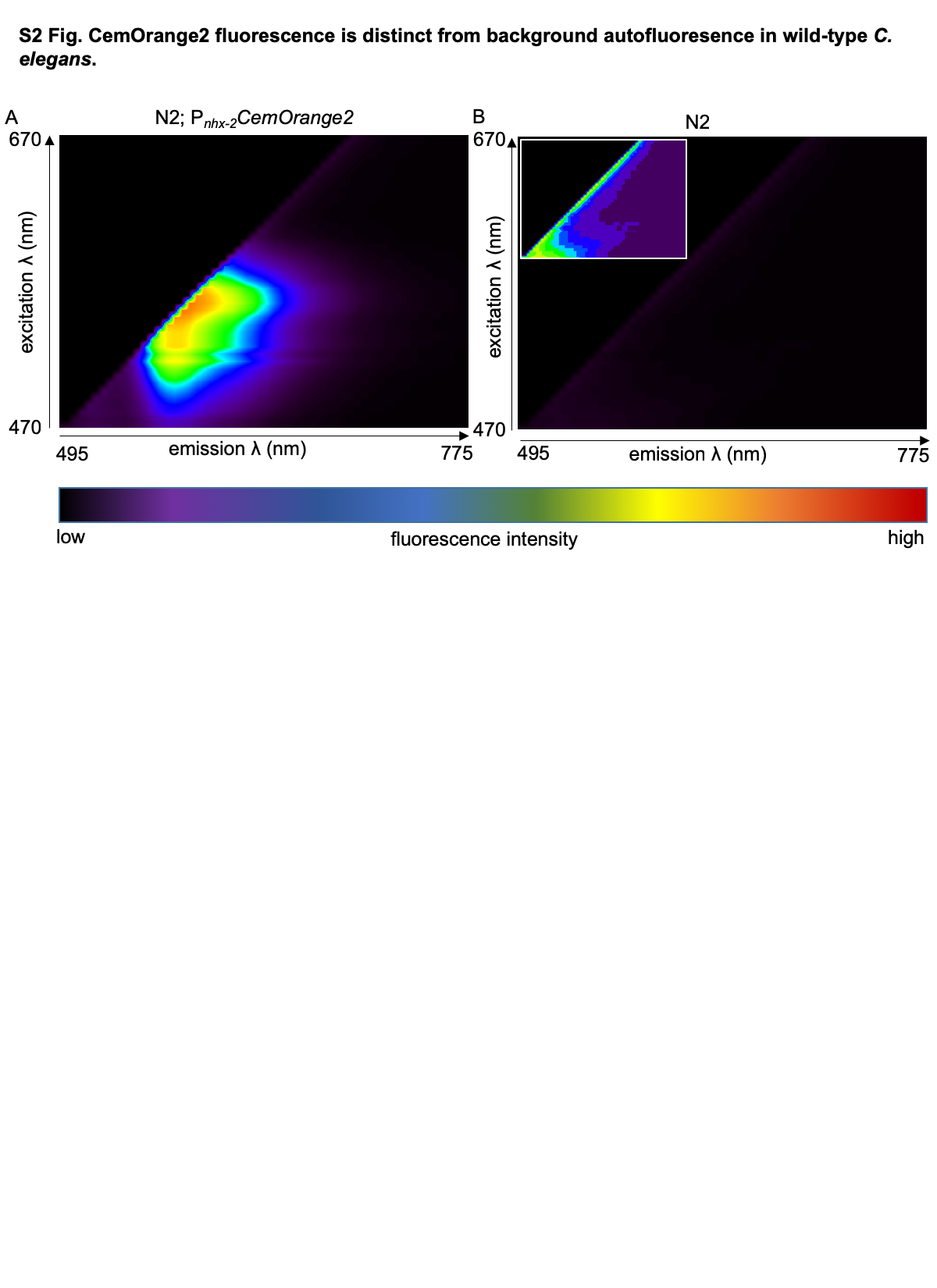

Supplement: S2 Fig — Either transgenic (A) Pnhx-2CemOrange2 or (B) wild-type (N2) C. elegans posterior intestines were imaged using a confocal microscope fitted with a white light laser and spectral detectors at varying excitation (range 470 nm-670 nm in 5 nm steps) and emission wavelengths (range 485–785 in 5 nm steps and a detection window of 20 nm). The fluorescence intensity (0–255) of each 8 bit image at each excitation and emission wavelength was plotted using the 2D bilinear excitation and emission lambda scan algorithm in the LASX software (Leica Microsystems, Buffalo Grove, IL) using the color gradient of fluorescence intensity indicated. The inset in B shows the autofluoresence of the wild-type C. elegans with a the look up table (LUT) rescaled to between 0 and 20 to show that autofluoresence is exhibited in the blue light range with minimal overlap with the CemOrange2 fluorescence spectrum. (TIFF) [file pone.0214257.s004.tiff]

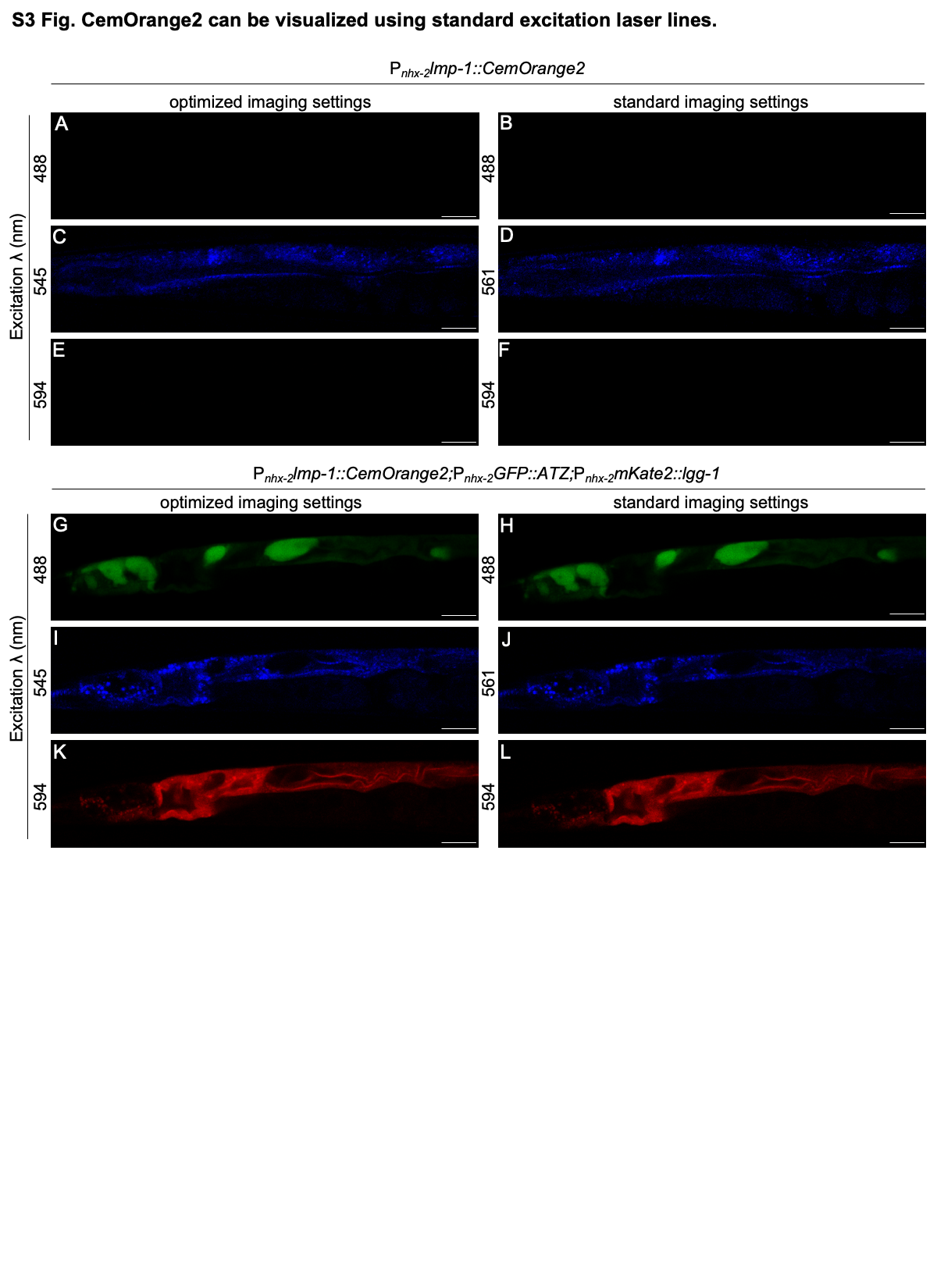

Supplement: S3 Fig — Either Pnhx-2lmp-1::CemOrange2 (A-F) or Pnhx-2lmp-1::CemOrange2;Pnhx-2GFP::ATZ;Pnhx-2mKate2::lgg-1 (G-L) transgenic C. elegans were imaged using a confocal microscope fitted with a white light laser set at either optimized 488, 545 and 594 nm (A, C, E, G, I, K) or standard 488, 561 and 594 nm (B, D, F, H, J, L) excitation wavelengths in sequential imaging mode over ≥30 z-planes. At either optimized or standard imaging excitation wavelengths, Pnhx-2lmp-1::CemOrange2 was detected only with the 545 nm (C; blue) or 561 nm (D; blue) excitation laser settings with similar punctate distribution with no cross talk with the 488 nm (A, B; green) and 594 nm (E, F; red) excitation laser lines. Pnhx-2lmp-1::CemOrange2;Pnhx-2GFP::ATZ;Pnhx-2mKate2::lgg-1 transgenic C. elegans imaged under the same conditions showed that all three fluorophores were readily detected using optimized or standard imaging settings; GFP::ATZ (G, H; green) using the Ex488 nm laser line, LMP-1::CemOrange2 (I, J; blue) with either Ex545 nm (I) and Ex561 nm (J) laser line and mKate2::LGG-1 (K, L; red) with the Ex594 nm laser line indicating that CemOrange2 can be utilized with more standard confocal imaging systems. Scale bar = 25 nm. (TIFF) [file pone.0214257.s005.tiff]
